# Expression and Functional Analyses of *Nymphaea caerulea* MADS-Box Genes Contribute to Clarify the Complex Flower Patterning of Water Lilies

**DOI:** 10.3389/fpls.2021.730270

**Published:** 2021-09-22

**Authors:** Silvia Moschin, Sebastiano Nigris, Ignacio Ezquer, Simona Masiero, Stefano Cagnin, Enrico Cortese, Lucia Colombo, Giorgio Casadoro, Barbara Baldan

**Affiliations:** ^1^Botanical Garden, University of Padua, Padua, Italy; ^2^Department of Biology, University of Padua, Padua, Italy; ^3^Department of Biosciences, University of Milan, Milan, Italy; ^4^CRIBI Biotechnology Center, University of Padua, Padua, Italy

**Keywords:** *Nymphaea caerulea*, flower development, MADS-box genes, flower evolution, ABCDE model, *AGAMOUS*, early diverging angiosperms, water lily

## Abstract

Nymphaeaceae are early diverging angiosperms with large flowers characterized by showy petals and stamens not clearly whorled but presenting a gradual morphological transition from the outer elements to the inner stamens. Such flower structure makes these plant species relevant for studying flower evolution. MADS-domain transcription factors are crucial components of the molecular network that controls flower development. We therefore isolated and characterized MADS-box genes from the water lily *Nymphaea caerulea*. RNA-seq experiments on floral buds have been performed to obtain the transcript sequences of floral organ identity MADS-box genes. Maximum Likelihood phylogenetic analyses confirmed their belonging to specific MADS-box gene subfamilies. Their expression was quantified by RT-qPCR in all floral organs at two stages of development. Protein interactions among these transcription factors were investigated by yeast-two-hybrid assays. We found especially interesting the involvement of two different *AGAMOUS-like* genes (*NycAG1* and *NycAG2*) in the water lily floral components. They were therefore functionally characterized by complementing Arabidopsis *ag* and *shp1 shp2* mutants. The expression analysis of MADS-box genes across flower development in *N. caerulea* described a complex scenario made of numerous genes in numerous floral components. Their expression profiles in some cases were in line with what was expected from the ABC model of flower development and its extensions, while in other cases presented new and interesting gene expression patterns, as for instance the involvement of *NycAGL6* and *NycFL*. Although sharing a high level of sequence similarity, the two *AGAMOUS-like* genes *NycAG1* and *NycAG2* could have undergone subfunctionalization or neofunctionalization, as only one of them could partially restore the *euAG* function in Arabidopsis *ag-3* mutants. The hereby illustrated *N. caerulea* MADS-box gene expression pattern might mirror the morphological transition from the outer to the inner floral organs, and the presence of transition organs such as the petaloid stamens. This study is intended to broaden knowledge on the role and evolution of floral organ identity genes and the genetic mechanisms causing biodiversity in angiosperm flowers.

## Introduction

The earliest angiosperm fossils date about 135 million years ago (MYA) (Willis and McElwain, 2013); however, it is now increasingly recognized that angiosperms originated before (van der Kooi and Ollerton, 2020). The exact timing of their origin is still under debate (Coiro et al., 2019; Li et al., 2019), whereas their great evolutionary success is not questioned, as angiosperms rapidly expanded to dominate most terrestrial habitats, today counting over 350,000 species (Scutt, 2018). The novelties of fruit and flower have undoubtedly contributed to the great success of flowering plants. Understanding the origin and rapid diversification of the angiosperm flower is a long-standing problem of evolutionary biology (Theiβen and Rümpler, 2018) in which Charles Darwin was also interested (letter of Darwin to JD Hooke, in Darwin, 2019). There are multiple reasons that make reconstruction of flower origin such a hard problem. Living gymnosperms and angiosperms form two sister clades whose lineages separated around 300 MYA, however the precise dating of the radiation of extant angiosperms is still uncertain. There are no living species that would link extant gymnosperms and angiosperms providing transitional forms of flower evolution (Bateman et al., 2006; Theiβen and Rümpler, 2018), and available unambiguous angiosperm fossils are not older than 130 MY (Sauquet et al., 2017). We are therefore facing a large morphological gap between extant gymnosperm and angiosperm reproductive structures, which makes it particularly difficult to trace back to the aspect of the earliest flowers. Furthermore, given the impressive radiation of angiosperms, hence the huge variation in their flower traits, it stands out the complexity in finding a comprehensive and thorough definition of the flower. From an evolutionary point of view, the angiosperm flower is a mix of novelties (Baum and Hileman, 2006). The carpel enclosing the ovules is considered one of the most relevant floral innovative structures (i.e., the feature that gave the group “angiosperms” their name). Then, other important flower-specific traits are bisexuality (male and female organs may be bore in the same axis) and the presence of a perianth surrounding the reproductive organs. The research field of flower evolution is very intriguing, not only because the conditions of flower appearance are still uncertain, but also because at the base of the huge variation of the existing floral morphologies there is a mostly conserved developmental genetic network for floral organ identity. During the last three decades, much has been learned about the genetic and molecular mechanisms underlying flower development, and MADS-box genes were identified as key regulators of floral organ identity. MADS-box genes are present in eukaryotes but are particularly numerous in seed plants, where some gene-family members are especially important for the development of reproductive structures (Gramzow and Theißen, 2013). Detailed studies of their role have mostly been performed in eudicot model plant species, where functional genetics approaches have elucidated many of their gene functions. During flower development, their specific functions depend on their well-defined expression domains in the different floral whorls. Based on data mostly from Arabidopsis, *Antirrhinum*, and *Petunia*, the genetic ABC model, later expanded into the ABCDE model, was proposed. In this model, class A + E genes specify sepals, A + B + E genes specify petals, B + C + E specify stamens, C + E specify carpels and C + D + E specify ovules (Theiβen et al., 2016, and references therein). This model constituted an important reference point for subsequent studies of flower development. Although the model was established based on eudicots, more recently it has been assessed outside eudicots and monocots (Theiβen and Rümpler, 2018). Studies in early diverging angiosperms (Buzgo et al., 2004; Kim et al., 2005; Soltis et al., 2007; Yoo et al., 2010) and in Ranunculales, a sister order to all other eudicots (Damerval and Becker, 2017 and references therein; Martínez-Gómez et al., 2021) evidenced both similarities and differences with respect to the ABCDE model.

In some early diverging angiosperms, such as water lilies (*Nymphaea* species), flowers are characterized by a large number of organs and gradual transitions in organ identity: from the outer elements to the inner ones outer sepaloid tepals are followed by petaloid tepals, petaloid stamens (or staminodes) and by stamens. The morphological gradation observed in flowers of these species has been linked to broader gene expression domains of the different functional classes, with respect to the ABCDE model (Buzgo et al., 2004; Kim et al., 2005; Yoo et al., 2010). Therefore, the “Fading Borders Model” has been proposed as a more suitable explanation for ANA grade (Amborellales, Nymphaeales, and Austrobaileyales) and Magnoliids flowers (Soltis et al., 2007). The Fading Borders Model has been discussed by Theissen and Melzer (2007) as a putative ancestral state of flower evolution, but it seemed not fitting quite well with the reconstruction made by Sauquet et al. (2017), which suggested that the ancestral flower likely presented whorled perianth and androecium. However, it is still quite debated whether the fading expression patterns of MADS-box genes involved in floral organ identity and the morphological gradation of floral organs are ancestral rather than evolved states in some lineages of early diverging angiosperms (Sauquet et al., 2017; Sokoloff et al., 2018; Scutt, 2018). Our research fits in these intriguing evolutionary questions and highlights the importance of studying MADS-box genes in non-model species placed in crucial phylogenetic positions. This work provides an extensive characterization of several MADS-box genes in *Nymphaea caerulea*. We evidenced both corroborative and novel results, with respect to the MADS-box genes expressed in the various floral organs, including in the transition elements that characterize the flower of this water lily. We found especially interesting the involvement of different genes belonging to the *AGAMOUS* subfamily. Compared with *Amborella*, which has only one *AG-like* gene and one *STK-like* gene (Amborella Genome Project, 2013), in *N. caerulea* we found two different *AG-like* genes (besides one *STK-like*). *NycAG1* and *NycAG2* resulted broadly expressed in floral organs, with partially overlapping expression profiles. With the aim of investigating whether these two paralogs could have specialized after duplication, we performed functional analyses by complementation experiments. The functional characterization of *NycAG1* and *NycAG2* in Arabidopsis *ag* and *shp1 shp2* mutants showed that only *NycAG1* could restore stamen and pistil development by complementation of the *ag-3* mutation, whereas neither *NycAG1* nor *NycAG2* complemented the *shp1 shp2* mutant.

## Materials and Methods

### *Nymphaea caerulea* Material, RNA Extraction, Quantification, and Cleaning

*Nymphaea caerulea* samples were collected from plants grown at the Botanical Garden of Padua, Italy (45.40797, 11.88586). We harvested floral buds at different stages of development during all the period from their emergence to flower anthesis. Floral buds have been dissected, the floral organs were observed under the stereomicroscope (Leica EZ4W). All the samples for RNA extraction were immediately frozen in liquid nitrogen and stored at −80°C. Total RNA was extracted from each sample as described in Lovisetto et al. (2012), quantified using a NanoDrop^®^ ND-1000 Spectrophotometer (Thermo Fisher Scientific), and treated with DNase I (NEB – New England Biolabs^®^) to remove genomic DNA. DNase I has been removed through the RNA Clean & Concentration^TM^-5 kit (Zymo Research). Total RNA has been quantified again and then conserved at −80°C until use.

### Pollen Viability Assay

Outer and inner stamens have been collected at different stages of development and pollen grains were collected in plastic tubes. Pollen viability was estimated using the Tetrazolium salt assay as described by Wang et al. (2004).

### Transcripts Isolation and Identification

**FIGURE 1:**
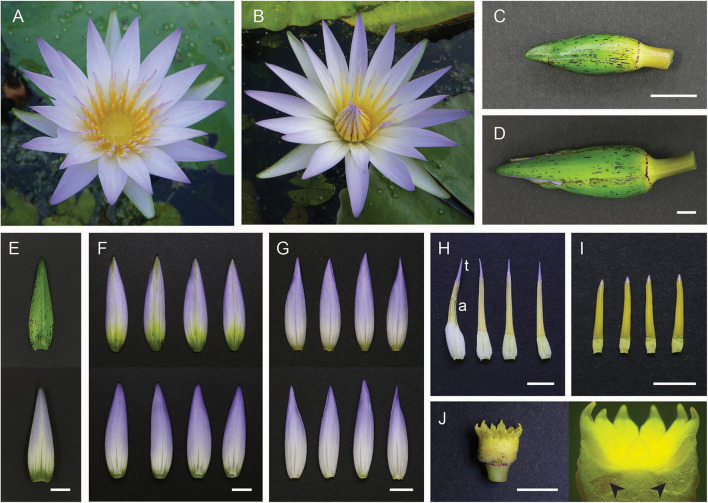
*Nymphaea caerulea* floral components. **(A)** Flower at first day of anthesis; **(B)** flower at second day of anthesis. **(C)** A total of 2.5 cm long floral bud; **(D)** 5.5 cm long floral bud. **(E)** Green abaxial surface (top) and whitish adaxial surface (bottom) of a sepal. **(F)** Abaxial surface (top) and adaxial surface (bottom) of outer petals. **(G)** Abaxial surface (top) and adaxial surface (bottom) of inner petals. **(H)** Petaloid stamens, “t” indicates the cerulean tip and “a” indicates the anther portion. **(I)** Inner stamens. **(J)** Pluricarpellate pistil. Arrowheads indicate ovules within carpels. Scale bar, 1 cm.

Two different RNA sequencing experiments were performed. We sequenced (i) RNA extracted from sepals, petals, stamens and carpels of four very young floral buds (1 cm long), and (ii) RNA extracted from carpels of three floral buds 2 cm long (see *N. caerulea* flower organs in Figure 1 and Supplementary Figure 1). For the first sequencing we extracted RNA from separated flower components in order to balance their contributions in the total amount of reads, as in the flower of *N. caerulea* tepal tissue is much more than other organ tissues. The second RNA-seq was performed to obtain a specific dataset from carpels. The first RNA-seq experiment was performed by myGenomics (Alpharetta, GA, United States), while the other one was performed by Novogene (Hong Kong). Both companies included a step of polyA enrichment in the library preparation protocol. Illumina sequencing generated 150 bp long paired-ended reads. Raw reads have been deposited in the NCBI database (PRJNA720641). Raw reads were quality filtered discarding: (i) reads with adaptor contamination; (ii) reads when uncertain nucleotides constitute more than 10 percent of either read (*N* > 10%); (iii) reads when low quality nucleotides (base quality less than 20) constitute more than 50 percent of the read. Quality filtered reads were *de novo* assembled by using Trinity software (software version r20140413p1; parameter min_kmer_cov: 2, others by default; Grabherr et al., 2011) to obtain transcriptome dataset (TSA submission SUB9462618). The annotation of the obtained transcripts was performed by comparison with seven databases (Nr, Nt, Pfam, KOG/COG, Swiss-Prot, KEGG, and GO). Transcript FASTA sequences obtained by the RNA-seq experiments were also analyzed by the online tool T-Rapid^1^ to identify MADS-box genes. The transcripts of interest were isolated, and their annotation was confirmed by BLASTn alignments. All the obtained sequences analyzed in this work have been deposited in the NCBI GenBank database and Accession Numbers are provided in Supplementary Table 1.

### Amplification by RT-PCR of *NycAP3* Isoforms

Putative transcriptional isoforms of *NycAP3* were obtained from the RNA-seq experiments. To exclude the possibility that these sequences were chimeric transcripts due to inaccurate reads assembly, we amplified them by RT-PCR. One microgram of total RNA extracted from floral buds was used for each cDNA synthesis reaction employing the SuperScript^TM^ III Reverse Transcriptase (Invitrogen) according to the manufacturer’s specifications. The sequence of the oligo d(T) used for mRNAs retrotranscription is reported in Supplementary Table 2. PCR reactions were set up in 25 μL using the WonderTaq (Euroclone) enzyme and following the product indications. Primer sequences (RT-PCR forward and reverse primers) are reported in Supplementary Table 2. In order to isolate as many as possible different sequences, degenerate forward oligonucleotides designed on the conserved MADS domain have been used (Supplementary Table 2). PCR products were sequenced and compared with the transcript sequences obtained by RNA-seq.

### Gene Expression Analysis by RT-qPCR

RT-qPCR was performed on flower samples collected from three floral buds at two different stages of development before anthesis: the first stage (early) consisted of buds about 2.5 cm long from tip to base (Figure 1C), and the second stage (late stage) consisted of buds about 5.5 cm long (Figure 1D). These two stages are representative of the developmental timeframe that ranges from when the tiny floral buds branch off from the central apex to flower anthesis. MADS-box gene expression levels were quantified in the floral organs reported in Figure 1, comparing the two developmental stages (Figure 1 and Supplementary Figure 1). Studied genes are listed in Supplementary Table 1. One microgram of total RNA extracted from each sample was used for each cDNA synthesis reaction employing the SuperScript^TM^ III Reverse Transcriptase (Invitrogen) according to the manufacturer’s specifications. RT-qPCR was performed in 10 μL using HOT FIREPol EvaGreen qPCR Mix Plus (Solys BioDyne). The experiments were conducted in a 7500 Real-Time PCR System (ThermoFisher). Gene-specific primers were designed by Primer-BLAST,^2^ and the specificity of each primer set was monitored by the analysis of the dissociation curves. Primer efficiency was calculated using serial dilutions of cDNA template. All primers have an efficiency from 80 to 100%. Actin transcript sequence (*NycACT*) has been used as the internal reference gene (Supplementary File 1). All the primer sequences are reported in Supplementary Table 2. The expression profiles of the analyzed genes have been reported in the graphs as initial RNA quantities calculated as 2^–ΔCt^. The experiments have been performed with three biological replicates and every qPCR reaction has been repeated three times (three technical replicates). Error bars in the graphs report the standard error of the mean (SEM).

### Phylogenetic Analysis

The isolated sequences were translated in the correct amino acidic sequences using the online ExPASy tool.^3^ Protein sequences belonging to the different MADS-box gene families were obtained from NCBI protein databases to be used as datasets to perform sequence alignments and phylogenetic analyses. Accession numbers of the considered sequences have been reported in the trees. Sequences were first aligned by ClustalW algorithm and then manually checked. The trees were generated using the PhyML package included in the software Seaview v. 4.7 (Gouy et al., 2010). The analyses were performed applying five random starts and 100 bootstrap replicates. The evolutionary distances were computed using the JTT matrix-based method and are in the units of the number of amino acid substitutions per site.

### Yeast Two-Hybrid Assays

Yeast two-hybrid (Y2H) assays were set up to evaluate protein–protein interaction using the yeast strain *Saccharomyces cerevisiae* AH109 (Clontech Laboratories, Palo Alto, CA, United States). The full-length and partial cDNAs have been cloned in Gateway-compatible variant of pGBKT7 and pGADT7 (see Supplementary Table 2; Daminato et al., 2014). All the yeast transformations were conducted using the highly efficient yeast lithium acetate transformation protocol (Schiestl and Gietz, 1989). For two-hybrid assays, transformants were initially selected for growth on media without tryptophan and leucine. Positive clones were subsequently tested on media without tryptophan, leucine, and adenine or histidine with increasing/different concentrations of 3-AT (3-aminotriazole, Sigma-Aldrich) and incubated at 22°C for about 5–7 days.

### Constructs and Arabidopsis Plant Transformation

Since water lily plants are currently not tractable to genetic transformation, we complemented known Arabidopsis class C mutants with *NycAG1* and *NycAG2*. Zhang et al. (2004) reported successful complementation of the Arabidopsis *ag* mutant using a gymnosperm sequence driven by the 35S constitutive promoter. However, to avoid potential pleiotropic effects caused by the use of strong constitutive promoters when working with MADS-box genes, we have used the Arabidopsis regulatory regions to perform the complementation experiments.

*Arabidopsis thaliana* plants were grown at 22°C under long-day conditions (LD). The mutants that have been used are *ag-3* (Ler) (Bowman et al., 1989) and *shp1 shp2* (Col) (Liljegren et al., 2000). *N. caerulea NycAG1* and *NycAG2* coding sequences have been cloned through BP recombination into pDONR 221 plasmid (ThermoFisher). The cloned coding sequences of *NycAG1* and *NycAG2* were inserted in the *pAG:GW* vector by LR recombination, generating respectively the *pAG:NycAG1* and *pAG:NycAG2*. We verified by sequencing that CDSs were in frame with *pAG* and downstream the whole intact chimeric *AGAMOUS* promoter. This promoter contains part of the second exon, the whole second intron region and the minimal 35S promoter and contains the necessary regulatory elements required to drive correctly *AtAG* expression (Busch et al., 1999; Hong et al., 2003). *NycAG1* and *NycAG2* were inserted in the *pSHP2:GW* vector (Ceccato et al., 2013; Balanzà et al., 2016) by LR recombination, generating respectively the *pSHP2:NycAG1* and *pSHP2:NycAG2*. We verified by sequencing that CDSs were in frame and downstream the SHP2 promoter. This promoter contains the 2154 bases upstream the *SHP2* transcriptional start site necessary to complement the *shp1 shp2* mutant fruit phenotype (Busch et al., 1999; Hong et al., 2003).

The GV3101 *Agrobacterium tumefaciens* strain was used for Arabidopsis transformation *via* the floral dip method (Clough and Bent, 1998). *Agrobacterium* strains carrying these constructs were used to transform *ag-3/*+ heterozygous plants of *A. thaliana* Ler. (T0), *shp1 shp2* and WT plants (as control). The seeds of these plants were harvested and the transformed plants were isolated through multiple rounds of BASTA selection in pots (spraying plants every 2 days for a total of six times). Transgenic lines carrying each specific transgene insertion were verified by PCR (primer sequences listed in Supplementary Table 2).

### Fixation, Clearing, and Phloroglucinol Staining of Arabidopsis Fruits

Arabidopsis siliques at stage 17 (Roeder and Yanofsky, 2006) were fixed in ethanol-acetic fixative (9:1) for 10 min under vacuum and kept overnight at 4°C. Samples were washed with 70% ethanol (30 min) and they were cleared in chloral hydrate:glycerol:H_2_O (8 g:1 mL:2 mL; w/v/v) solution for 24 h. Samples were dehydrated in a series of five increasing ethanol concentrations. Finally, Arabidopsis fruits were stained with 2% phloroglucinol solution in 96% ethanol for 5 min and then 50% HCl was applied for 1 min. Dissected fruits were then analyzed by Zeiss Axiophot D1 microscope equipped with differential interface contrast (DIC) optics and images were recorded with an Axiocam MRc5 camera (Zeiss) using the Axiovision program.

### Quantification of *NycAGs* Expression in Arabidopsis Transformed Lines by RT-qPCR

For *pAG:NycAG1* and *pAG:NycAG2* expression analyses, *A. thaliana* RNA from floral buds of the primary inflorescence were collected after the bolting. For *pSHP:NycAG1* and *pSHP:NycAG2* expression analyses, *A. thaliana* inflorescences at 15a and 15c developmental stages (Roeder and Yanofsky, 2006) were pooled separately and collected. Total RNA was extracted using the LiCl method and converted into first-strand cDNA through the ImProm-II Reverse Transcription System (Promega, Madison, WI, United States). The expression levels of the transgenes were evaluated by RT-qPCR assay with a Bio-Rad iCycler iQ optical system (software version 3.0a). For each sample, RT-qPCR was conducted in two different biological replicates and three technical replicates. Diluted aliquots of the first-strand synthesis were used as templates in the RT-qPCR reactions containing the iQ SYBR Green Supermix (Bio-Rad). The primers used for quantification experiments are listed in Supplementary Table 2. Arabidopsis *ACTIN 8* (RT861-RT862) gene was used as an internal reference. The expression levels of *NycAG1* and *NycAG2* were evaluated using the 2^–ΔΔCt^ method (Livak and Schmittgen, 2001).

## Results

### The Flower Structures of *Nymphaea caerulea*

*Nymphaea caerulea* is a rhizomatous tropical water lily. Leaves and flowers branch off from a submerged mixed central apex, according to a growth pattern that gradually moves away the older structures from the apex, allowing the growth of younger shoots and buds. The floating leaves have a slightly oval shape and grow on long petioles inserted in the center of the lamina, thus allowing the plant to cover a large water surface. The hermaphroditic flower of *N. caerulea* arises above the water surface one day before anthesis thanks to the growth of the petiole. Like most of water lily flowers, this flower is protogynous with a diurnal opening. The first day of anthesis stamens are in a vertical position (Figure 1A), facilitating the access of pollinators to the exposed pistil, which is surrounded by a stigmatic fluid that attracts insects (Schneider and Chaney, 1981); during the first day of anthesis, the flower is thus prepared to receive pollen from other flowers. From the day after, the flower keeps the stamens in an oblique position, concealing almost completely the stigma (Figure 1B). Therefore, pollinators must go across stamens to reach the nectar, covering themselves with the pollen produced by the mature male organs. This mechanism promotes cross-fertilization. *N. caerulea* floral organs are shown in Figure 1. The flower is characterized by a star shape (Figure 1A) and a morphological transition from the outermost elements to the innermost stamens. The bud (Figures 1C,D) is enclosed by four laminar elements that have a green abaxial surface and a contrasting whitish adaxial surface (Figure 1E), whereas all other “tepals” are cerulean and resemble proper attractive elements (Figures 1F,G). We named the first “sepals” and the other “petals.” Such distinction was also suggested by Yoo et al. (2010) for *Nymphaea odorata*. Proceeding toward the center of the flower there are numerous stamens. A marked morphological gradation can be noticed from the outermost petaloid stamens (Figure 1H) to the smaller inner ones (Figure 1I). Petaloid stamens have a cerulean distal tip, whereas the part immediately below is a yellowish anther with four pollen sacs that produce viable pollen grains similar to the anthers of inner stamens. Finally, at the center of the flower, there is a pluricarpellate pistil (Figure 1J). Each carpel contains many ovules (indicated with arrowheads in Figure 1J).

### MADS-Box Genes Isolated in *Nymphaea caerulea* Developing Flowers

MADS-domain transcription factors are important components of the regulatory network that controls the development of reproductive structures in land plants (reviewed in Pajoro et al., 2014). To identify floral organ identity MADS-box genes in *N. caerulea* two independent RNA-seq experiments using RNA from different young flowers (see section “Materials and Methods”) were performed. From these experiments, we obtained about 3.59 × 10^8^ raw pair-ended reads that resulted in 2.33 × 10^8^ quality-filtered reads. From the *de novo* assembled transcriptome dataset (NCBI SUB9462618), we obtained a total of 122,819 contigs, 122,778 of which were identified as putative gene transcripts. Supplementary Table 1 summarizes the flower MADS-box genes identified in the transcript datasets.

**FIGURE 2:**
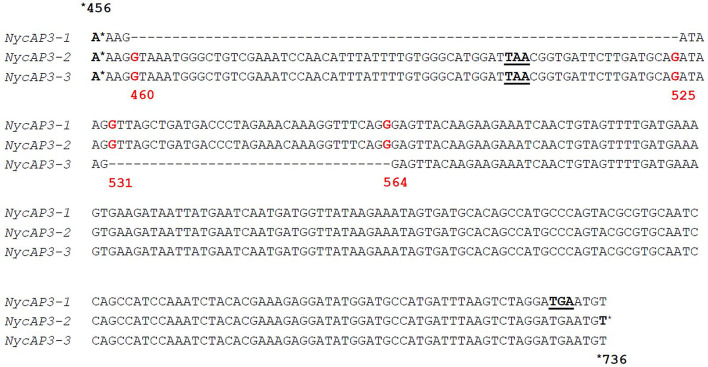
Partial alignment of the three *NycAP3* transcripts isolated in this work and annotated as *NycAP3-1*, *NycAP3-2*, and *NycAP3-3*. The alignment shows the regions where the differences among the three sequences are situated. STOP codons are underlined. Red numbers refer to nucleotide positions on the *NycAP3-2* sequence from the start ATG. The alignment was built in accordance with Stellari et al. (2004).

One *NycFUL-like* (*NycFL*) transcript was identified, and phylogenetic analysis of the deduced protein showed that it formed a clade with known AP1/FUL sequences from other Nymphaeaceae (Supplementary Figure 2). Consistent with the complexity of sepals, petals, and stamen structures, we identified several B-type sequences: one *NycPI* and three different *NycAP3* transcripts. Stellari et al. (2004) identified three different *AP3-like* sequences in a *Nymphaea* species and proposed that they derived from alternative splicing. Based on the sequence comparisons, we have also hypothesized that the three *N. caerulea AP3* sequences might be splicing variants (*NycAP3-1*, *NycAP3-2*, and *NycAP3-3*) of the same *AP3* gene (Figure 2). Sequence identity between *NycAP3-1* and *NycAP3-2* is of 99.78%, between *NycAP3-1* and *NycAP3-3* is of 94.47% and between *NycAP3-2* and *NycAP3-3* the sequence identity is of 95.97%. In particular, *NycAP3-1* lacks a 66-bp fragment between nucleotides 460 (numbered from the start ATG) to nucleotide 525. This fragment is present in the other two splicing variants and includes a stop codon (Figure 2); thus, the *NycAP3-2* and *NycAP3-3* sequences might both be translated into the same (shorter) protein. On the contrary, the *NycAP3-1* sequence might produce a longer protein. Notably, the cDNA sequences of *NycAP3-2* and *NycAP3-3* differed from each other downstream from the stop codon, because *NycAP3-3* lacked a 34-bp fragment (from nucleotide 531 to 564) (Figure 2). Consistent with the above data, phylogenetic analysis of B-type proteins evidenced that NycAP3-2/NycAP3-3 form a small clade with the AP3 sequences of class II and III identified by Stellari et al. (2004), whereas NycAP3-1 belongs to a different clade (Supplementary Figure 3). Since it has been reported that in the *Nymphaea colorata* genome there is a single *AP3* gene (Zhang et al., 2020), it might be the same for other *Nymphaea* species. As expected, NycPI grouped within the PISTILLATA clade, together with other water lily PI sequences (Supplementary Figure 3).

Three different *AGAMOUS-like* (*NycAG1, NycAG2*, and *NycSTK*) transcripts were also retrieved. Sequence identity between *NycAG1* and *NycAG2* is of 74.73%, between *NycAG1* and *NycSTK* is of 78.72% and between *NycAG2* and *NycSTK* the sequence identity is of 73.10%. Phylogenetic analysis (Supplementary Figure 4) showed that NycSTK grouped with other SEEDSTICK-like sequences, whereas NycAG1 and NycAG2 clustered with other water lily AGAMOUS-like sequences (Shan et al., 2009). Only one *SEP* transcript (named *NycSEP*) was identified from *N. caerulea* floral buds, and phylogenetic analysis showed that NycSEP grouped with other Nymphaeaceae sequences and that this clade was included in the AGL2 super-clade (Supplementary Figure 5). We also identified a single *AGL6* gene (*NycAGL6*) (Supplementary Figure 6).

### Analysis of MADS-Box Gene Expression in the Flower Organs of *N. caerulea*

MADS-box gene expression was quantified in flower organs sampled from floral buds still submerged corresponding to two different stages of development. The early stage consisted of buds 2.5 cm long from tip to base (Figure 1C), while the late stage consisted of buds 5.5 cm long (Figure 1D). The organs that have been analyzed were sepals (Figure 1E and Supplementary Figure 1A), outer petals (larger) (Figure 1F and Supplementary Figure 1B), inner petals (smaller) (Figure 1G and Supplementary Figure 1C), petaloid stamens (cerulean tips and anthers separately) (Figure 1H and Supplementary Figure 1D), and inner stamens (Figure 1I and Supplementary Figure 1E), and carpels, with ovules removed and analyzed separately (Figure 1J and Supplementary Figure 1F).

**FIGURE 3:**
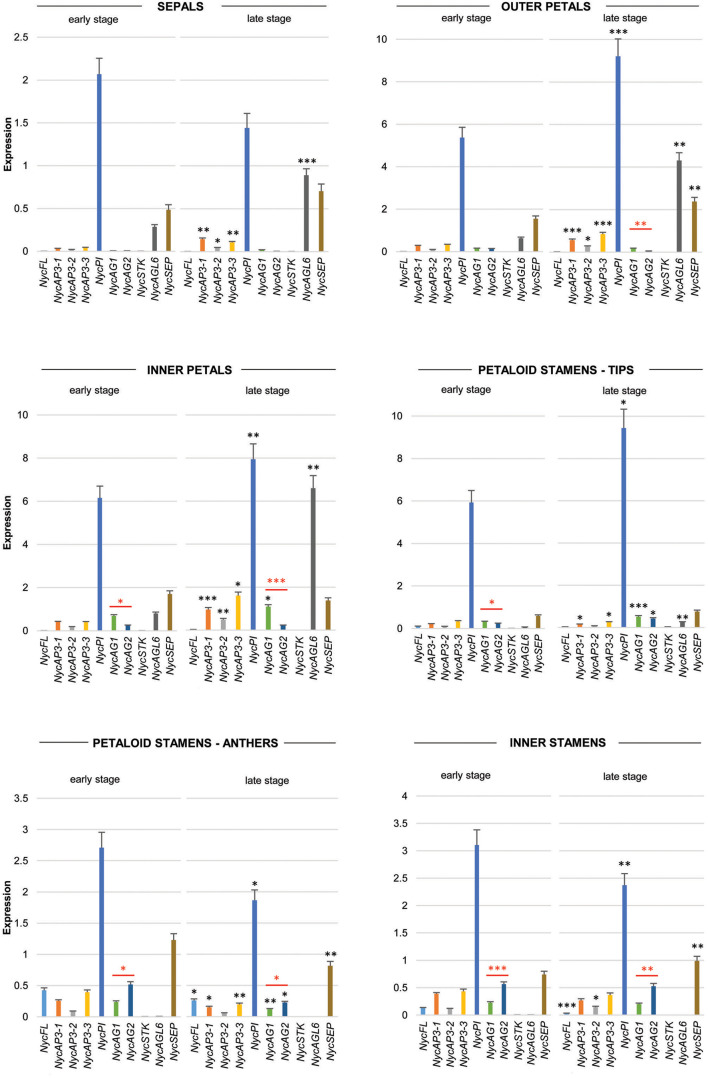
Analysis of MADS-box gene expression during sepals, petals, and stamens development. Expression profiles of MADS-box genes in floral components (sepals, petals, petaloid stamens, and inner stamens) obtained from floral buds at two different stages of development (early and late stages, respectively) by qPCR experiments. Values in the graphs represent initial RNA quantities ± SEM. Black asterisks above an expression level indicate a significant difference between the two developmental stages, for that specific gene. Red asterisks indicate a significant difference of the expression levels of genes under the red bar (^∗^*p* < 0.05; ^∗∗^*p* < 0.01; ^∗∗∗^*p* < 0.001).

Real-time PCR data (Figure 3) showed that *NycFL, NycAG1, NycAG2*, and *NycSTK* were not expressed in sepals. The three *NycAP3* isoforms were weakly expressed, but expression increased significantly in sepals at the late stage. By contrast, *NycPI* was robustly transcribed, especially in the youngest sepals. *NycSEP* and *NycAGL6* were weakly expressed in sepals, and their expression was slightly higher in the oldest sepals. No *NycFL* and *NycSTK* expression was detected in inner and outer petals, whereas all other genes resulted expressed and displayed higher transcript levels in older petals compared to the younger ones. In particular, *NycPI* and *NycAGL6* transcripts strongly accumulated during petal development. *NycAG1* and *NycAG2* were expressed in petals, with *NycAG1* showing higher expression than *NycAG2*, but neither gene was expressed in sepals. Moreover, the expression of *NycAG1* was higher in inner petals than in the outer ones (*p* = 0.01 early stage; *p* = 0.0002 late stage). *NycFL* was expressed in anthers, with stronger expression at the early stage. *NycPI* was transcribed in anthers and in petaloid cerulean tips and expression increased at a late stage, similar to inner petals. In anthers, the highest *NycPI* expression was observed at the early stage. *NycAGL6* was poorly expressed in the petaloid cerulean tips, and no expression was detected in any anther type. *NycSEP* was detected in all tissues, but the highest expression was observed in both anther types. As expected, both *NycAG1* and *NycAG2* were expressed in anthers. No *NycSTK* expression was detectable in both petaloid and inner stamens.

**FIGURE 4:**
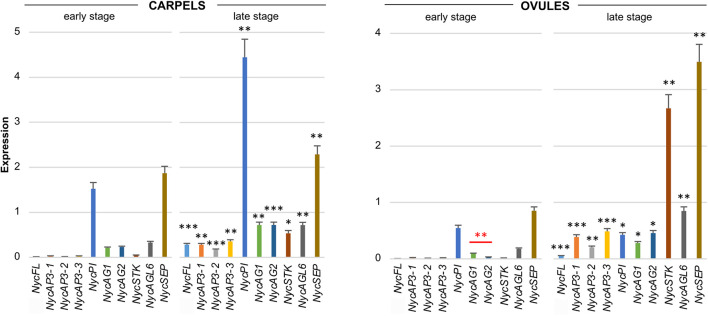
Analysis of MADS-box gene expression during carpels and ovules development. Expression profiles of MADS-box genes obtained from carpels and ovules at two different stages of development (early and late stages) of the floral bud. The expression profiles were obtained by qPCR experiments; values in the graphs represent initial RNA quantities ± SEM. Black asterisks above an expression level indicate a significant difference between the two developmental stages, for that specific gene. Red asterisks indicate a significant difference of the expression levels of genes under the red bar (^∗^*p* < 0.05; ^∗∗^*p* < 0.01; ^∗∗∗^*p* < 0.001).

All the MADS-box genes analyzed were expressed in carpels and, in general, their transcription increased at the late development stage (Figure 4). In the youngest organs, *NycPI* and *NycSEP* displayed the strongest expression, while *NycAG1, NycAG2*, and *NycAGL6* were also expressed at a lower level. At older stages, all genes showed increased expression (Figure 4). In very young ovules, all genes except for *NycSEP* and *NycPI* were expressed at very low levels. The overall expression levels increased in older ovules, especially *NycSEP* (fourfold increase) and *NycSTK* (more than 170-fold).

### Analysis of MADS-Domain Protein–Protein Interactions by Yeast Two-Hybrid Assays

An extensive matrix-based Y2H screen involving many Arabidopsis MADS-domain transcription factors demonstrated that MIKC proteins interact preferentially with other type-II proteins in forming the floral quartet model (see the review of Theiβen et al., 2016).

We therefore tested the potential physical interactions among the MADS-domain transcription factors identified in *N. caerulea*. These analyses demonstrated that NycPI can heterodimerize with either NycAP3-1 and NycAP3-2/NycAP3-3, as expected for B-type MADS-domain proteins (Egea-Cortines et al., 1999). We also tested whether the B-type transcription factors could interact with NycAG1, NycAG2, or NycSTK, but we failed to detect the formation of dimers among any B- and C-type MADS-domain factors (Table 1). Similarly, NycPI does not heterodimerize with NycSEP or NycFL, whereas NycAP3-1 and NycAP3-2/NycAP3-3 interact with NycSEP although quite weakly, indeed yeast colonies were grown at 22°C and with none or very few 3-AT (1 mM). A truncated version of NycSEP lacking part of the C-terminus region was used to prevent the activation of the reporter genes without the contribution of any NycSEP interactors (Amborella Genome Project, 2013). Previous studies had shown that SEP proteins are relatively promiscuous in their interactions (Immink et al., 2009). As expected, NycSEP could heterodimerize with NycAG1, NycAG2, NycSTK, NycFL, and NycAGL6. Furthermore, NycFL could heterodimerize with the two *Nymphaea* AG proteins and with NycSTK, but was unable to interact with either NycAP3-1 or NycPI alone. NycAGL6 was demonstrated to interact with NycSEP, NycAG1, and NycAG2 but not with NycSTK; in addition, NycAGL6 was unable to form dimers with the B-type transcription factors (Table 1).

**TABLE 1:** Results of yeast two-hybrid assays for *N. caerulea* MADS-domain proteins.

AD	NycSEP	NycFL	NycAG1	NycAG2	NycSTK	NycAP3-1	NycAP3-2/3	NycPI	NycAGL6
BD									
NycSEPΔ	n.t.	++	++	++	++	±	±	±	++
NycFL	++	n.t.	++	++	++	−	−	−	n.t.
NycAG1	++	++	n.t.	−	−	−	−	−	++
NycAG2	++	++	−	n.t.	−	−	−	−	++
NycSTK	++	++	−	−	n.t.	−	−	−	−
NycAP3-1	±	−	−	−	−	n.t.	n.t.	+	−
NycAP3-2/3	±	−	−	−	−	n.t.	n.t.	+	−
NycPI	+	−	−	−	−	+	+	n.t.	−
NycAGL6	++	n.t.	++	++	−	−	−	−	n.t.

*Interactions have been scored on selective medium lacking tryptophan, leucine, adenine and histidine with 0 or 1 mM 3-AT. GAL4BD-MADS proteins do not activate the reporter gene transcription without an interacting protein partner. NycSEPΔ lacks part of the C-terminus to prevent the activation of the reporter genes without the contribution of NycSEP interactors. The strength of the interactions has been visually evaluated scoring the results of at least three independent transformations: ++ is robust growth, + is growth, ± is poor growth, − is no growth, and n.t. is not tested.*

### Functional Analysis of *NycAG1* and *NycAG2* by Complementation Experiments With Arabidopsis *ag* and *shp1 shp2* Mutants

*AGAMOUS* subfamily members typically promote stamen and carpel identity as well as floral meristem determinacy (Kramer et al., 2004). We decided to investigate in more detail the *NycAG-like* genes for different reasons. Firstly, carpel represents a key morphological innovation and *AGAMOUS* plays a crucial role in its development process. Secondly, we found two different *AG-like* genes (*NycAG1* and *NycAG2*) expressed in the water lily floral tissues, while *Amborella* has only one *AG-like* gene, and we found this result particularly interesting. Furthermore, the expression analysis showed us that *NycAG1* and *NycAG2* share similar expression profiles during flower development, albeit with some differences. Therefore, we deepened the investigation on their putative functional specialization.

**FIGURE 5:**
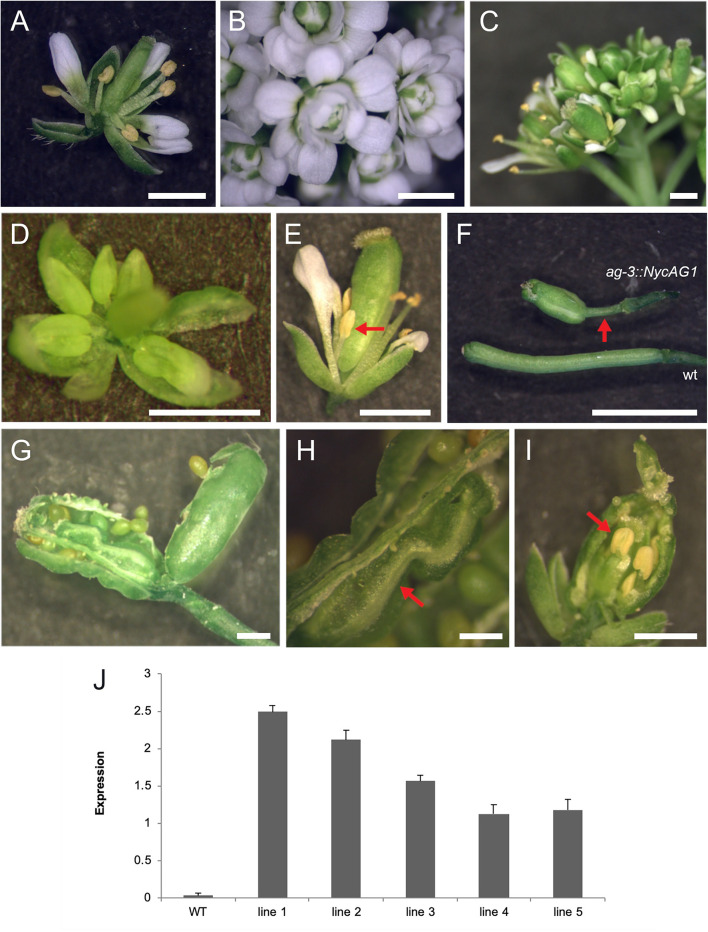
*NycAG1* can complement the Arabidopsis *AGAMOUS* function. **(A)** Wild-type flower. **(B)** Flower of an *ag-3* line carrying *pAG:NycAG2* showing a clear “not complemented” *ag-3* phenotype. **(C–E)** Flowers from transgenic line 3 plants of Arabidopsis *ag-3* carrying *pAG:NycAG1* displayed recovery of stamens (marked with red arrows) and functional pistils. **(F–I)** Functional fruits were formed upon pollination from transgenic line 3 plants of Arabidopsis *ag-3* carrying *pAG:NycAG1*. **(F)** Fruits presented very long gynophores respect to wild-type (marked with red arrow). **(G–I)** Inside the fruits, functional seeds were present which were able to germinate later. Those fruits presented also internally other floral organs like carpels **(H)** and stamens **(I)** (marked with red arrow). Bars in **(A–I)**, 1 mm. **(J)** qPCR analysis showed that *pAG:NycAG1* was expressed in floral buds of the transformed lines. Values in the graph represent initial RNA quantities ± SD.

We introduced *NycAG1* and *NycAG2* into the Arabidopsis *ag-3−/−* loss-of-function mutant under the control of the *AG* regulatory region (Sieburth and Meyerowitz, 1997; Deyholos and Sieburth, 2000). The flowers of the *ag-3* mutant are indeterminate and consist of reiterating sepal, petal, petal whorls (Bowman et al., 1991). Because *ag-3* mutant plants are sterile, *ag-3/*+ plants were independently transformed with *pAG:NycAG1* and *pAG:NycAG2* constructs. For *NycAG1*, 20 T1 plants were genotyped and five independent transgenic *ag* mutants containing the *pAG:NycAG1* construct and expressing *NycAG1* were analyzed in detail (Figures 5A–J). The *pAG:NycAG1* construct could partially rescue the *ag-3* flower phenotype in all the five lines. The complemented *ag-3* plants were able to produce flowers with stamens in the third whorl and pistils in the fourth whorl (Figures 5C–E). The pistils appeared quite abnormal, with a longer gynophore with respect to the wild-type (Figure 5F). Occasionally extra carpels (Figures 5G,H) and stamens developed from the placenta inside the primary carpel (Figure 5I). The stigma might also show defective phenotype such as lack of stigmatic papillae. The stamens of the transgenic plants produced viable pollen. The seeds produced by the fertilization were viable. Overall our results indicate that *NycAG1* can partially overcome *AG* absence regarding floral organ identity but it frequently not overcomes the floral meristem determination.

A similar experiment was performed by transforming *ag-3/*+ mutants with the *pAG:NycAG2* construct. Out of six *ag-3* homozygous plants that were expressing *NycAG2*, none could rescue the *ag-3* flower phenotype (Figure 5B and Supplementary Figure 7), suggesting that *NycAG2* cannot complement *AtAG* function.

In Arabidopsis, the *SHP1* and *SHP2* gene paralogs also belong to the *AGAMOUS* subfamily. Siliques of the Arabidopsis *shp1 shp2* double mutant fail to differentiate the valve margin and the fruits are indehiscent (Roeder et al., 2003; Balanzà et al., 2016). We transformed *shp1 shp2* with the *pSHP2:NycAG1* and *pSHP2:NycAG2* constructs, using 2154 bases upstream from the *SHP2* transcriptional start site as *SHP2* regulative region, since *pSHP2:SHP2* can complement the *shp1 shp2* mutant fruit phenotype (Balanzà et al., 2016). We obtained eight independent transgenic lines for *NycAG1* and three independent lines for *NycAG2*. Despite being expressed (Supplementary Figure 8), *pSHP2:NycAG1* and *pSHP2:NycAG2* constructs both failed to complement *shp1 shp2*, because the characteristic valve margin tissue did not differentiate in transgenic plants (Supplementary Figure 9A), the lignification of which can contribute to silique shattering (Liljegren et al., 2000) (Supplementary Figure 9B). Wild-type fruits showed lignification of the valve margin cells adjacent to the dehiscence zone throughout the siliques, but no staining was observed in *shp1 shp2* plants carrying the *pSHP2:NycAG1* or *pSHP2:NycAG2* constructs (Supplementary Figure 9C).

## Discussion

Nowadays the need to investigate floral organ identity genes in early diverging angiosperms has become increasingly important to expand the knowledge across angiosperm phylogeny and to help to make reconstructions of the earliest flowers. Species belonging to the ANA-grade group (Amborellales, Nymphaeales, and Austrobaileyales) are of great interest not only because they constitute the earliest bifurcations in the phylogeny of angiosperms, but also because their flowers are characterized by a great diversity of structure and form. Both large, multipartite bisexual flowers, and small, simple, frequently unisexual flowers can be found, and variation in the number and arrangement of floral parts is extremely high (Friis et al., 2011). It is reasonable to consider that this great morphological variation among early diverging flowers is the outcome of a flexible genetic control of the flower organization.

**FIGURE 6:**
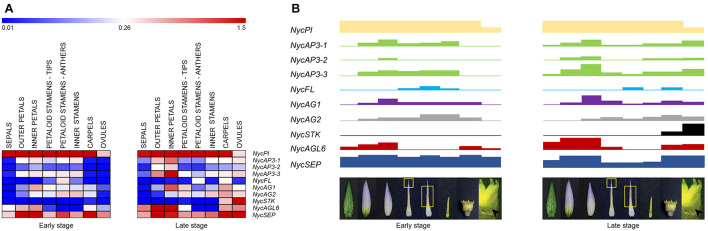
MADS-box gene expression in the various floral components and flower developmental model in *Nymphaea caerulea*. **(A)** Heat-map of the MADS-box gene expression levels at two developmental stages (early and late, respectively) of flower development. Expression values were scaled by 2^–ΔCt^ obtained by qPCR experiments. **(B)** Block expression model showing the contribution of each gene in the tissues analyzed (from left to right: sepals, outer petals, inner petals, cerulean tips of petaloid stamens, anthers of petaloid stamens, inner stamens, carpels, and ovules). Rectangle heights from **(A)**.

In most studied eudicot species, MADS-box transcription factors involved in flower development have been divided into functional classes described by the ABCDE model (see section “Introduction”). In *N. caerulea*, the expression patterns of MADS-box genes isolated so far mirror the high morphological complexity of the flower (Figure 6). In other words, we characterized a model of gene expression in which many different floral organ identity genes are involved (more B and C class genes, *AGL6*, *FUL-like*, *SEP*). Most of the genes showed a broad and continuous expression pattern in different floral components, while others had a more discrete profile considering neighboring flower organs. The results obtained in this work confirm that the genetic model suitable for eudicot flowers is not strictly applicable to this species. The broader and overlapping expression profiles of many genes can be responsible for the observed gradual transition of floral organ morphology. This scenario appears to be common in *Nymphaea* species, strengthening the assumptions of Yoo et al. (2010) and Zhang et al. (2020). Even though studying a non-model species was challenging, this choice yielded important positive implications. Providing a deep expression analysis considering two stages of flower development and a functional characterization of the two *NycAGs*, this study was able to highlight some novelties concerning the involvement of the various MADS-box in water lily flowers.

### The Array of MADS-Box Genes in the Flower of *Nymphaea caerulea*

The *NycFL* gene was detected only in *N. caerulea* carpels and anthers. Zhang et al. (2020) also reported that a *FUL-like* gene was expressed in stamens and carpels in *N. colorata*. The analysis of several AP1/FUL protein sequences (Litt and Irish, 2003; Shan et al., 2007) revealed the presence in all the sequences of a FUL motif (Shan et al., 2007), of two conserved motifs in the in the C-terminus portions of euAP1 proteins (farnesylation and euAP1 motifs, Litt and Irish, 2003), and a conserved FUL-like motif in the euFUL and FUL-like proteins (Litt and Irish, 2003; Shan et al., 2007). The NycFL protein sequence contains either the FUL either the FUL-like motifs (Supplementary Figure 10) and therefore it can be considered a FUL-like protein, similarly to the related proteins from other Nymphaeales (*Nuphar advena* and *Cabomba caroliniana*) (Kim et al., 2005; Yoo et al., 2010). This supports the reconstructed *AP1/FUL* history, which describes the *FUL-like* genes as pre-duplication genes, present in ANA grade species and non-core eudicots (Litt and Irish, 2003; Pabón-Mora et al., 2012).

A single *PISTILLATA* gene (*NycPI*) and three different *AP3* transcripts (*NycAP3-1*, *NycAP3-2*, and *NycAP3-3*) were identified in *N. caerulea*. Stellari et al. (2004) isolated several *AP3* sequences from a *Nymphaea* sp. and reported that different AP3 proteins could be produced *via* alternative splicing. Yoo et al. (2010) also identified several *AP3-like* sequences from *N. odorata*. The hypothesis that a single *AP3-like* gene might be the progenitor of the different splicing variants observed in various water lilies (Stellari et al., 2004; Yoo et al., 2010; this work) has been recently confirmed by the analysis of the *N. colorata* genome which seems to contain only one *AP3-like* gene (Zhang et al., 2020). *NycPI* was highly transcribed in all floral organs; its expression markedly increased in old petals as well as in old cerulean tips of petaloid stamens. In contrast, it decreased in anthers of petaloid stamens and in inner stamens, at late stages. In addition, also *NycAGL6*, *NycAG1*, and *NycAG2* showed similar expression profiles comparing petals and cerulean tips of petaloid stamens. Moreover, *NycAGL6* expression appeared peculiar and interesting as it was confined to sepals, petals and cerulean tips of petaloid stamens, but it was absent in anthers. These results suggest that the two cerulean structures can share some similar developmental patterns. The petaloid stamens represent a transitional structure between inner stamens and petals. In general, petals derive from modifications of either bracts (bracteopetals) or stamens (andropetals) (Ronse de Craene and Brockington, 2013). The detailed expression analysis in *N. caerulea* reinforces the idea proposed by Yoo et al. (2010) that petals of Nymphaeaceae species “originated from petaloid staminodes”; therefore *N. caerulea* petals should be considered as “andropetals.”

SEP and AGL6 proteins belong to two separate clades that are part of a common super-clade (Zahn et al., 2005; Rijpkema et al., 2009). Until now *SEP* genes have been evidenced only in angiosperms where their number can vary in different species. For instance, two *SEP* genes are present in the genome of *Amborella* (Amborella Genome Project, 2013) whereas eight *SEP-like* members were identified in the genome of apple (Ireland et al., 2013). In *N. caerulea* transcripts of only one *SEP* and one *AGL6* gene have been isolated from the overall reproductive structures, confirming data obtained from the *N. colorata* genome (Zhang et al., 2020). The involvement of *NycAGL6* in the perianth was in line with previous data that attributed an A function to *AGL6* in some species. For instance, the characterization of an *AGL6* gene in *Petunia* (*PhAGL6*) showed that it redundantly controlled petal and anther development with two *SEP* genes (*FBP2* and *FBP5*) (Rijpkema et al., 2009). Accordingly, both the expression analysis of *AGL6* in *Aristolochia fimbriata* (Pabón-Mora et al., 2015; Peréz-Mesa et al., 2020) and the functional analysis of *AGL6* in *Nigella damascena* (Wang et al., 2015) further suggested that this gene plays a role in perianth identity. Also in *N. colorata* Zhang and collaborators assign an A function to *AGL6* as it appeared expressed at high levels only in sepals and petals, while in *N. caerulea NycAGL6* was found expressed also in carpels and ovules at late developmental stages. This interesting finding further extends the discussion about the A function. Indeed, the concept of A function has been considered controversial for almost as long as the ABC model was proposed (Theissen et al., 2000; Litt, 2007; Causier et al., 2010). Further studies are required to characterize more deeply the putative functions of *AGL6* genes in flower development.

### The *AGAMOUS* Subfamily in *Nymphaea caerulea*

Phylogenetic analysis of the three *N. caerulea* AGAMOUS-like protein sequences revealed that one of them (NycSTK) clustered with other STK-like sequences. In particular, it is close to a water lily sequence already defined as STK-like by Shan et al. (2009). In accordance with this analysis, *NycSTK* was expressed in ovules, showing a dramatic increase in the late stage, which suggests its crucial importance for ovule development. As regards the other two *AG-like*, *NycAG1*, and *NycAG2* shared a similar expression pattern even if the two profiles were slightly shifted because *NycAG1* expression was higher in petals, while that of *NycAG2* in stamens. Moreover, their decreasing expression toward the outer region of the flower is consistent with a “fading borders” model (Buzgo et al., 2004; Soltis et al., 2007; Theissen and Melzer, 2007; Soltis et al., 2009). Yoo et al. (2010) also identified two *AG-like* genes and a *STK-like* gene in another water lily species (*N. odorata*) and found that the *STK-like* gene was highly expressed in ovules, whereas the two *AG-like* genes were expressed in all other floral organs. Expression data presented in Figure 6 show that there is a general expression increment in the ovules and carpels in the late stage of development. This stage is closer to flower anthesis, and probably the flower is getting ready for receiving the pollen and for the subsequent fertilization. Altogether, it seems that expression data obtained from late stage flowers of *N. caerulea* might resemble the expression data presented in Zhang et al. (2020), where the developmental stage of the analyzed flower is not reported. Since the genome of *Amborella trichopoda* contains only one *AGAMOUS-like* gene (Amborella Genome Project, 2013), while water lilies have two *AGAMOUS-like* genes (Yoo et al., 2010; Zhang et al., 2020; this study) and both of them are expressed in flower organs, it appeared highly interesting to functionally characterize them in *ag-3* and *shp1 shp2* Arabidopsis mutants. The ability of the sole *NycAG1* to partially restore the wild-type flower phenotype in the Arabidopsis *ag-3* mutant suggests that the paralogs *NycAG1* and *NycAG2* act significantly differently, reflecting a possible subfunctionalization or neofunctionalization. On the other hand, none of the two *NycAGs* was able to rescue *shp1 shp2* mutants in Arabidopsis, and a possible explanation may be linked to the fact that *SHATTERPROOF* genes have specialized after the duplication of *AG/PLE* genes occurred in core eudicots. Subfunctionalization of AGAMOUS genes has also been studied in a non-core eudicot species (i.e., the ranunculid *Thalictrum thalictroides*) (Galimba et al., 2012; Galimba and Di Stilio, 2015). The functional characterization of the two *AG-like* genes of *T. thalictroides* evidenced that even belonging to the same lineage the two paralogs have undergone subfunctionalization, since one of them (*ThtAG1*) is working as a C-function gene (Galimba et al., 2012), while the other one (*ThtAG2*) has acquired D function (Galimba and Di Stilio, 2015).

These findings highlight that in early diverging angiosperms and more in general in non-core eudicot species, a great level of complexity in MADS-box transcription factors can be found.

### Protein*–*Protein Specific Interactions Between MADS-Box of *Nymphaea caerulea*

Yeast two-hybrid analysis has been extensively employed to establish the high order complexes formed by MADS-domain transcription factors (Davies et al., 1996; Theiβen and Saedler, 2001; de Folter et al., 2005; Daminato et al., 2014). As expected, the results of this assay indicated that NycAP3 could heterodimerize with NycPI, in agreement with previous data (Egea-Cortines et al., 1999; Melzer et al., 2014), although, at least in yeast, these proteins interacted less strongly than other MADS-box dimers. In core eudicots, the formation of the AP3-PI dimer is needed to strongly settle their own expression, thus ensuring that both proteins are formed only in those cells where both transcripts are present (Schwarz-Sommer et al., 1992; Goto and Meyerowitz, 1994). Such feature was also retrieved for the B-type proteins of *A. trichopoda* and *N. advena* (Melzer et al., 2014). Interestingly *N. advena* AGL6 can heterodimerize with PISTILLATA, which might be in agreement with its putative involvement as A class protein (Honma and Goto, 2001). However, we did not detect this interaction in *N. caerulea*, similarly to what has been observed by Melzer et al. (2014) in *A. trichopoda*. It is possible that a third partner, such as the widely expressed NycSEP and/or A class proteins, might be necessary to form a higher order complex with AP3 and PI (Egea-Cortines et al., 1999; Honma and Goto, 2001). The NycSEP protein exhibited the typical features of the clade because it could heterodimerize with all MADS-domain proteins tested except NycPI. NycAGL6 showed specific interactions because it could dimerize with NycAG1 and NycAG2 but not with NycSTK. Altogether, results in yeast confirm that the NycMADS-domain proteins may form a complex network of protein–protein interactions that are in agreement with previous observations, although we could identify some features typical of this species.

## Conclusion

This work provides an extensive and general view of the MADS-box genes expressed during flower development in *N. caerulea*, highlighting some peculiarities of the architecture of water lily flowers. One such characteristic is the presence of two *AG-like* genes named *NycAG1* and *NycAG2*, which showed similar expression profiles, even if they seem to have acquired some functional specialization, as it appeared that only one of them could partially restore the Arabidopsis *AG* function in *ag-3* mutants. Another peculiarity pertains to *NycAGL6*, which has not been included in a wider flower development model so far, even though it appears to be important, as also demonstrated by data presented in this work. An interesting expression profile was also found for *NycFL* which resulted expressed in stamens and carpels. Finally, the expression of only one *SEPALLATA* gene (*NycSEP*) in all floral organs is in accordance with data of the *N. colorata* genome recently published (Zhang et al., 2020).

## Data Availability Statement

The datasets presented in this study can be found in online repositories. The names of the repository/repositories and accession number(s) can be found below: https://www.ncbi.nlm.nih.gov/, PRJNA720641.

## Author Contributions

BB and GC conceived and designed the work. SMo and SN carried out most of the experimental work with the help of IE and LC for complementation experiments in Arabidopsis, SMa for yeast two-hybrid experiments, SC for design and interpretation of qPCR experiments, and EC for *N. caerulea* sampling and part of the molecular biology experiments. GC, SMo, SN, and BB wrote the manuscript. IE, LC, SMa, EC, SC, read, revised, and approved the manuscript. All authors have agreed to the submitted version of the manuscript.

## Conflict of Interest

The authors declare that the research was conducted in the absence of any commercial or financial relationships that could be construed as a potential conflict of interest.

## Publisher’s Note

All claims expressed in this article are solely those of the authors and do not necessarily represent those of their affiliated organizations, or those of the publisher, the editors and the reviewers. Any product that may be evaluated in this article, or claim that may be made by its manufacturer, is not guaranteed or endorsed by the publisher.

## Footnotes

1
http://bioinformatics.psb.ugent.be/webtools/trapid

2
www.ncbi.nlm.nih.gov/tools/primer-blast/

3
https://web.expasy.org/translate/
