# Supplementary material for: Expression and Functional Analyses of Nymphaea caerulea MADS-Box Genes Contribute to Clarify the Complex Flower Patterning of Water Lilies
Source: Front Plant Sci. 2021 Sep 22;12:730270. doi: 10.3389/fpls.2021.730270 (PMC8492926; doi:10.3389/fpls.2021.730270)
Supplement: Supplementary file 1 [file Data_Sheet_1.PDF]

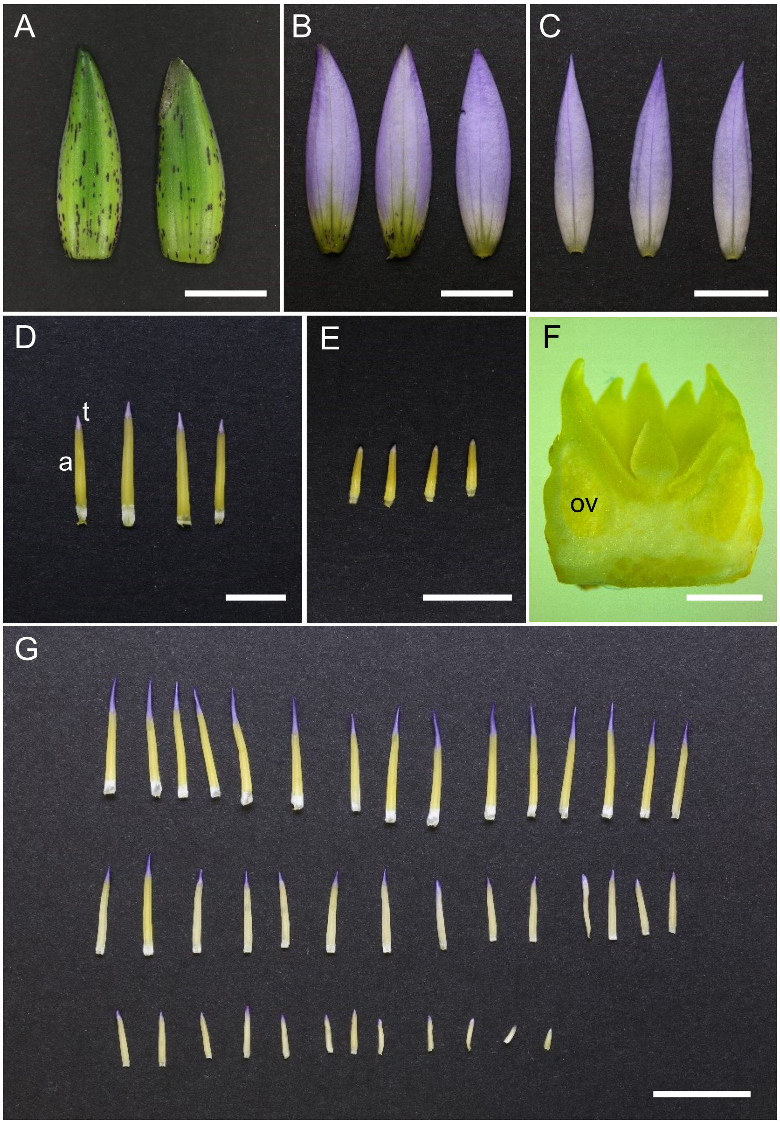

Supplementary Figure 1. *Nymphaea caerulea* floral elements of young floral buds. From (**A**) to (**F**): flower organs of an early stage bud 2.5 cm long. (**A**) Sepals; (**B**) outer petals; (**C**) inner petals; (**D**) petaloid stamens ("t" = tip, "a" = anther); (**E**) inner stamens; (**F**) carpels; "ov" = ovules inside carpels. (**G**) All the stamens that are contained in a 2 cm long floral bud, to show stamens morphological gradation. Scale bars in from (**A**) to (**E**) and in (**G**) = 1 cm; bar in (**F**) = 2 mm.
