# Supplementary material for: Expression and Functional Analyses of Nymphaea caerulea MADS-Box Genes Contribute to Clarify the Complex Flower Patterning of Water Lilies
Source: Front Plant Sci. 2021 Sep 22;12:730270. doi: 10.3389/fpls.2021.730270 (PMC8492926; doi:10.3389/fpls.2021.730270)
Supplement: Supplementary file 4 [file Data_Sheet_4.PDF]

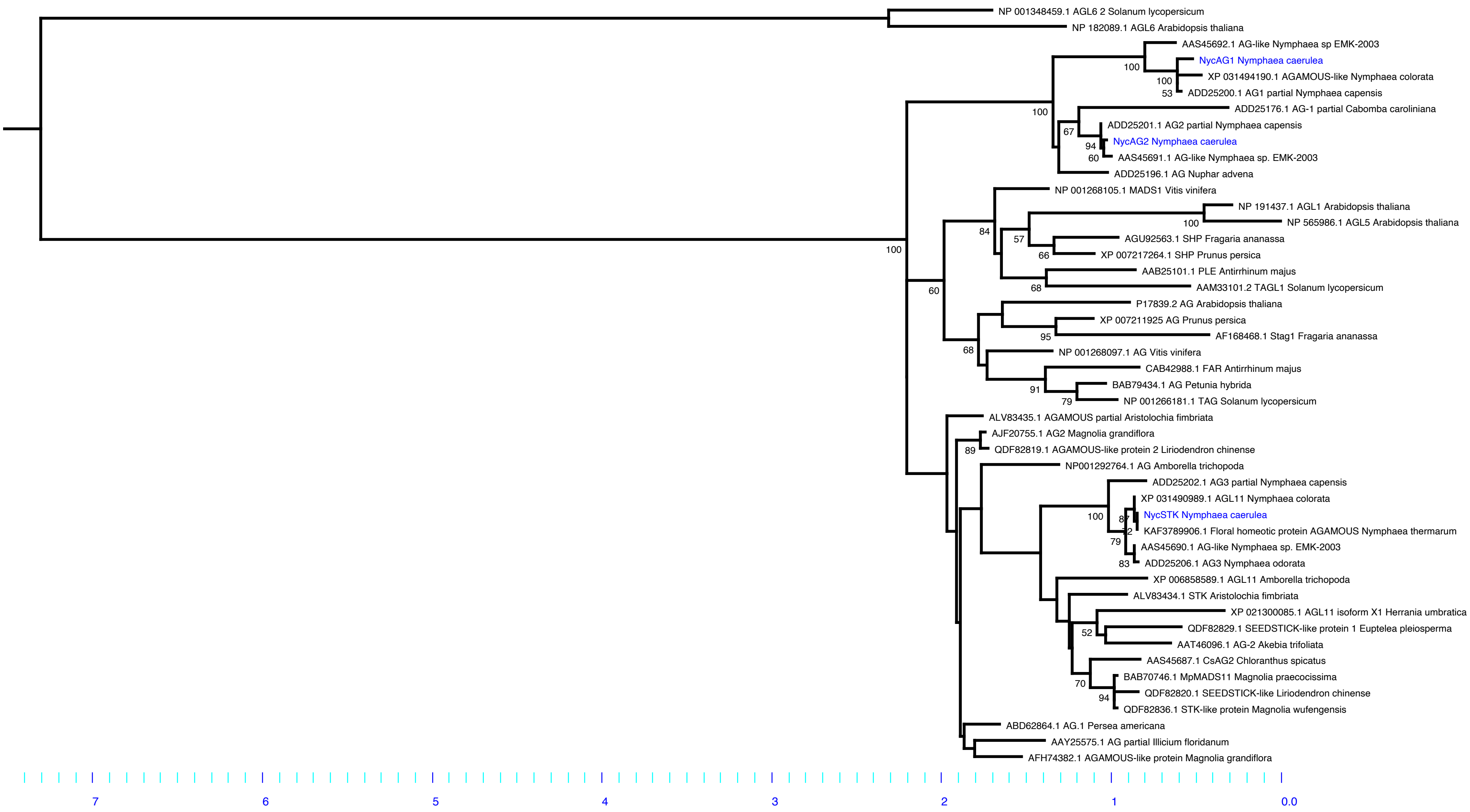

Supplementary Figure 4. Maximum-likelihood tree of 42 representatives of C and D lineage protein sequences plus the NycAG1, NycAG2 and NycSTK proteins. The tree has been generated using the PhyML package included in the software Seaview v. 4.7. The analysis was performed applying 5 random starts and 100 bootstrap replicates. The evolutionary distances were computed using the JTT matrix-based method and are in the units of the number of amino acid substitutions per site. The *N. caerulea* sequences are evidenced in blue. The tree has been rooted using AGL6 protein sequences.
