# Supplementary material for: Expression and Functional Analyses of Nymphaea caerulea MADS-Box Genes Contribute to Clarify the Complex Flower Patterning of Water Lilies
Source: Front Plant Sci. 2021 Sep 22;12:730270. doi: 10.3389/fpls.2021.730270 (PMC8492926; doi:10.3389/fpls.2021.730270)
Supplement: Supplementary file 5 [file Data_Sheet_5.PDF]

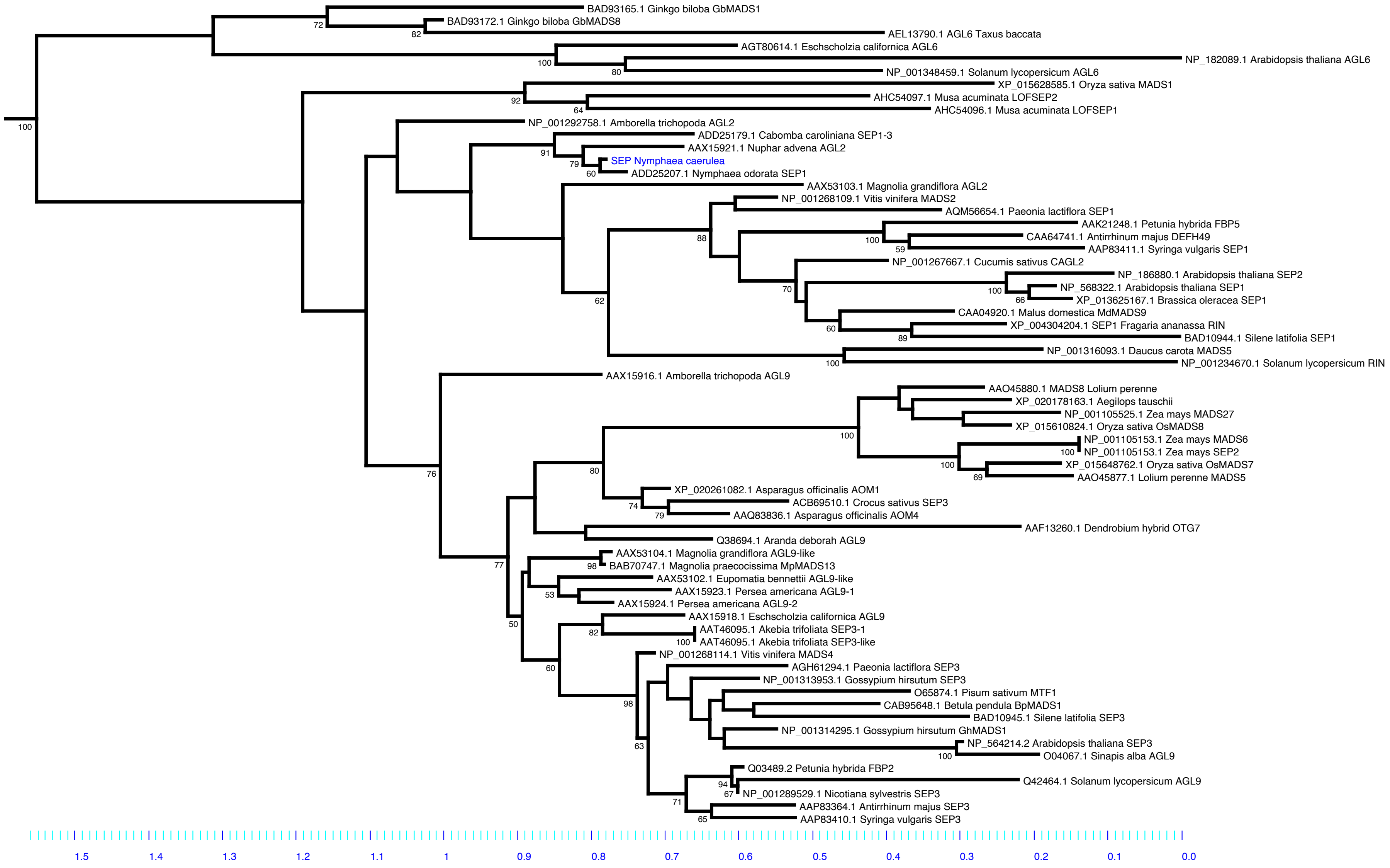

Supplementary Figure 5. Maximum-likelihood tree of 58 representatives of E lineage protein sequences plus the NycSEP protein. The tree has been generated using the PhyML package included in the software Seaview v. 4.7. The analysis was performed applying 5 random starts and 100 bootstrap replicates. The evolutionary distances were computed using the JTT matrix-based method and are in the units of the number of amino acid substitutions per site. *N. caerulea* sequence is evidenced in blue. The tree has been rooted using AGL6 protein sequences.
