# Supplementary material for: Expression and Functional Analyses of Nymphaea caerulea MADS-Box Genes Contribute to Clarify the Complex Flower Patterning of Water Lilies
Source: Front Plant Sci. 2021 Sep 22;12:730270. doi: 10.3389/fpls.2021.730270 (PMC8492926; doi:10.3389/fpls.2021.730270)
Supplement: Supplementary file 6 [file Data_Sheet_6.PDF]

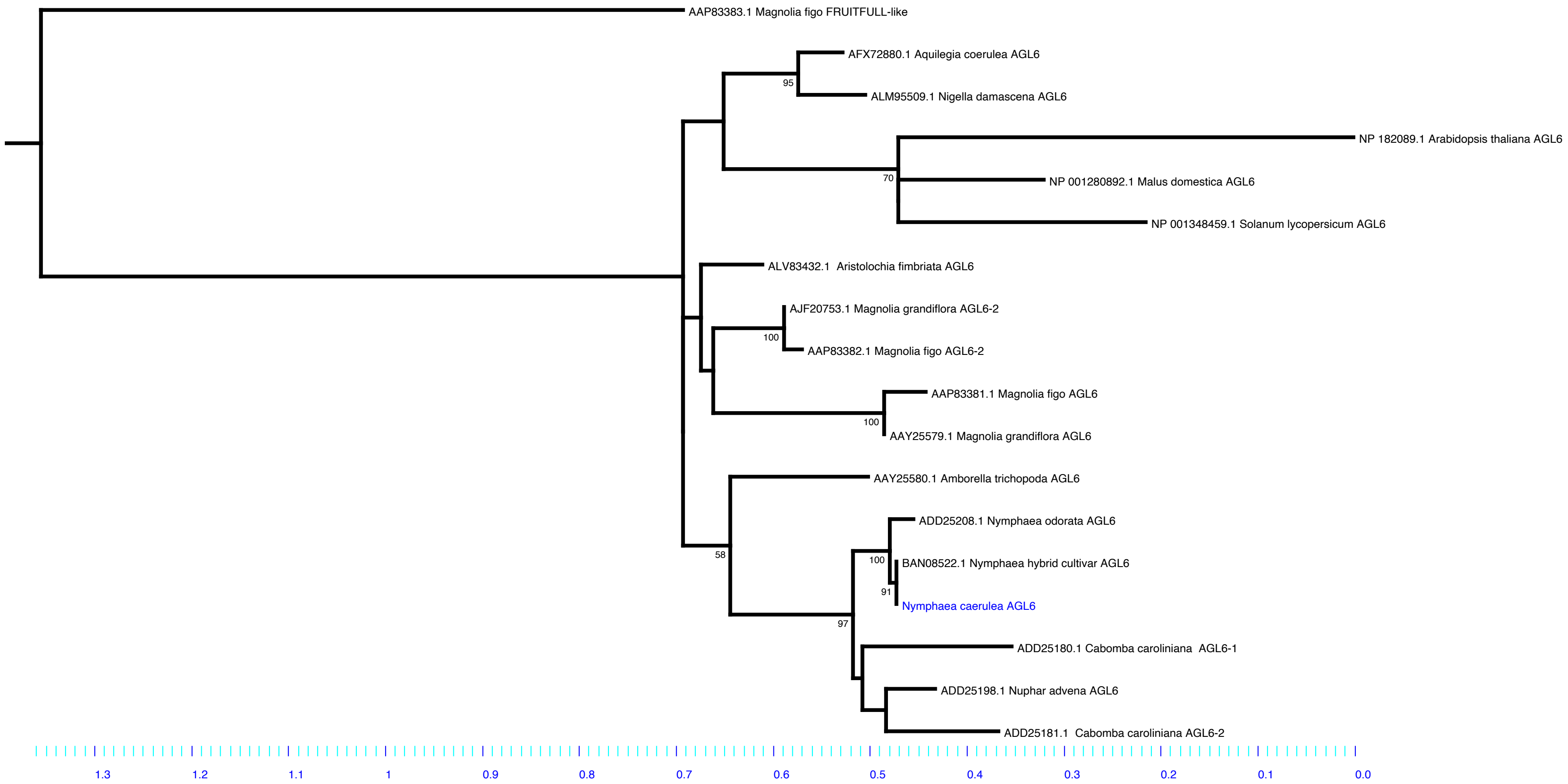

Supplementary Figure 6. Maximum-likelihood tree of 16 representatives of AGL6 protein sequences plus the NycAGL6 protein. The tree has been generated using the PhyML package included in the software Seaview v. 4.7. The analysis was performed applying 5 random starts and 100 bootstrap replicates. The evolutionary distances were computed using the JTT matrix-based method and are in the units of the number of amino acid substitutions per site. *N. caerulea* sequence is evidenced in blue. The tree has been rooted using a FUL protein sequence.
