# Supplementary material for: Expression and Functional Analyses of Nymphaea caerulea MADS-Box Genes Contribute to Clarify the Complex Flower Patterning of Water Lilies
Source: Front Plant Sci. 2021 Sep 22;12:730270. doi: 10.3389/fpls.2021.730270 (PMC8492926; doi:10.3389/fpls.2021.730270)
Supplement: Supplementary file 7 [file Data_Sheet_7.PDF]

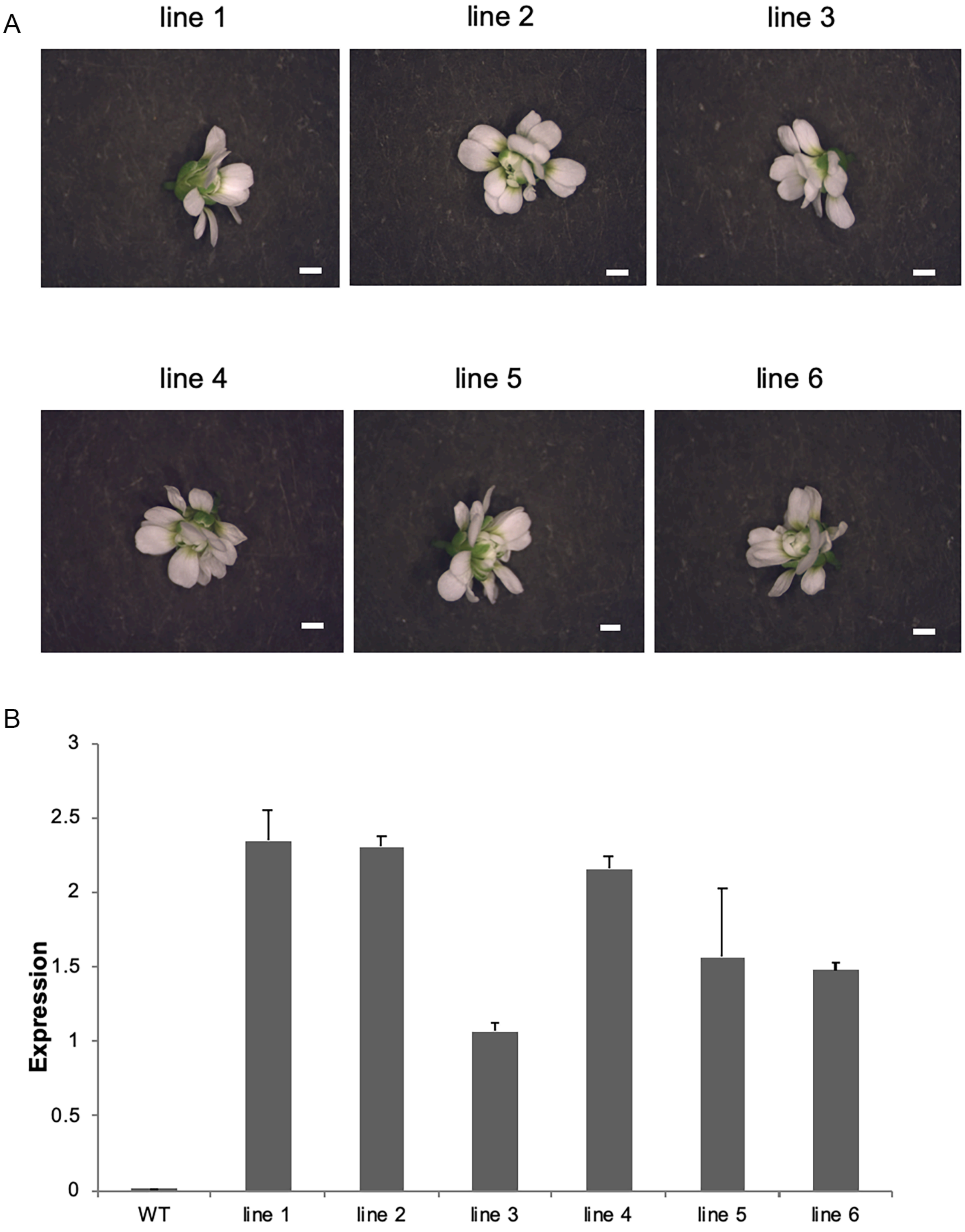

Supplementary Figure 7. Flowers of *ag*-3 plants expressing *NycAG2*.
(**A**) Homeotic conversion of floral organs into petals in *ag*-3 plants expressing *NycAG2*. (**B**) Expression levels of the *NycAG2* transgene in the analysed lines. Values in the graph represent initial RNA quantities ± standard deviation.
