# Supplementary material for: Expression and Functional Analyses of Nymphaea caerulea MADS-Box Genes Contribute to Clarify the Complex Flower Patterning of Water Lilies
Source: Front Plant Sci. 2021 Sep 22;12:730270. doi: 10.3389/fpls.2021.730270 (PMC8492926; doi:10.3389/fpls.2021.730270)
Supplement: Supplementary file 8 [file Data_Sheet_8.pdf]

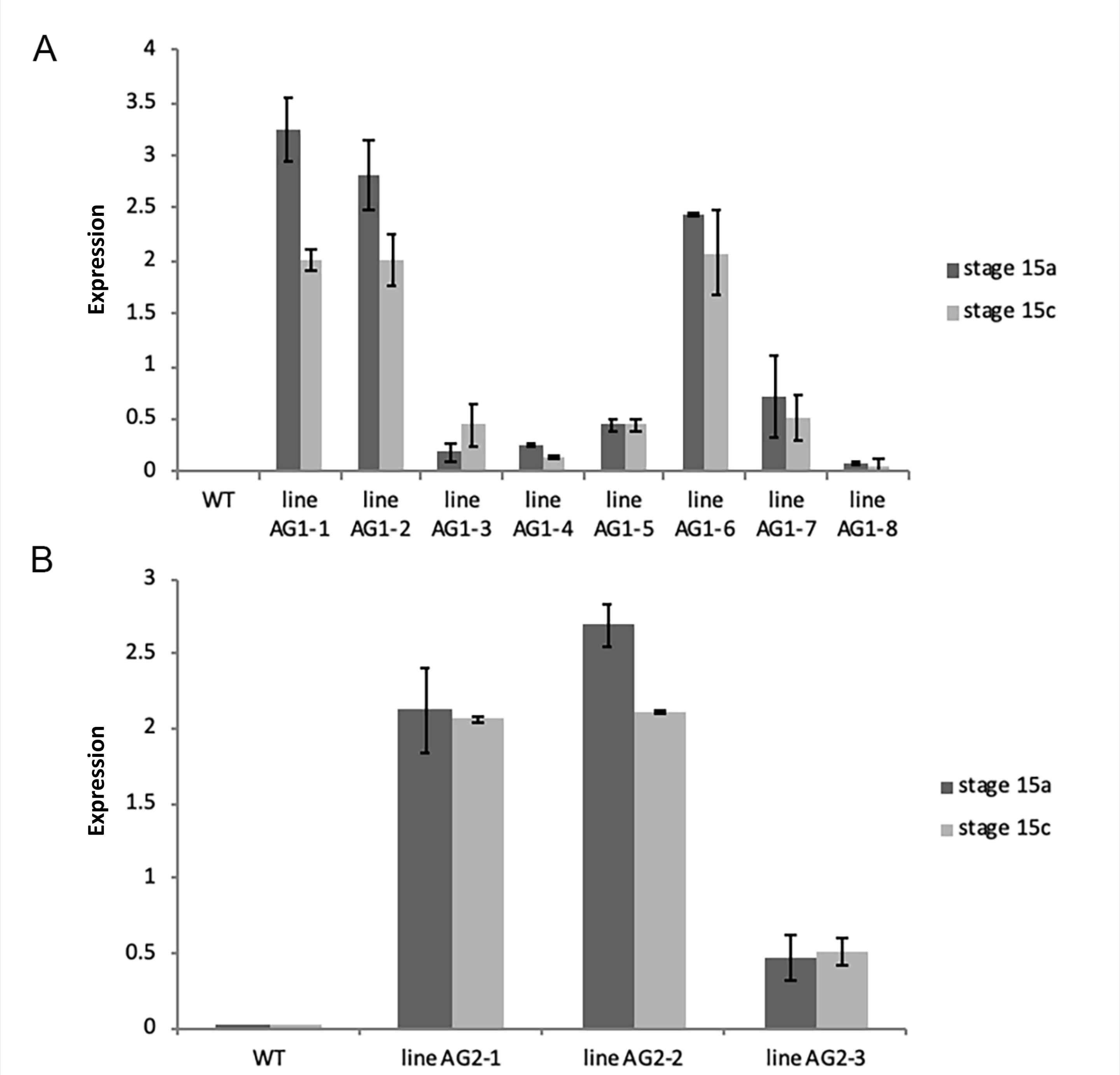

Supplementary Figure 8. Expression levels of *NycAG1* and *NycAG2* in *shp1 shp2 Arabidopsis* lines. (**A**) Expression *of pSHP2:NycAG1* and (**B**) expression of *pSHP2:NycAG2* in two developmental stages of the fruit (stage 15a and stage 15c). Values in the graphs represent initial RNA quantities ± standard deviation.
