# Supplementary material for: Expression and Functional Analyses of Nymphaea caerulea MADS-Box Genes Contribute to Clarify the Complex Flower Patterning of Water Lilies
Source: Front Plant Sci. 2021 Sep 22;12:730270. doi: 10.3389/fpls.2021.730270 (PMC8492926; doi:10.3389/fpls.2021.730270)
Supplement: Supplementary file 9 [file Data_Sheet_9.PDF]

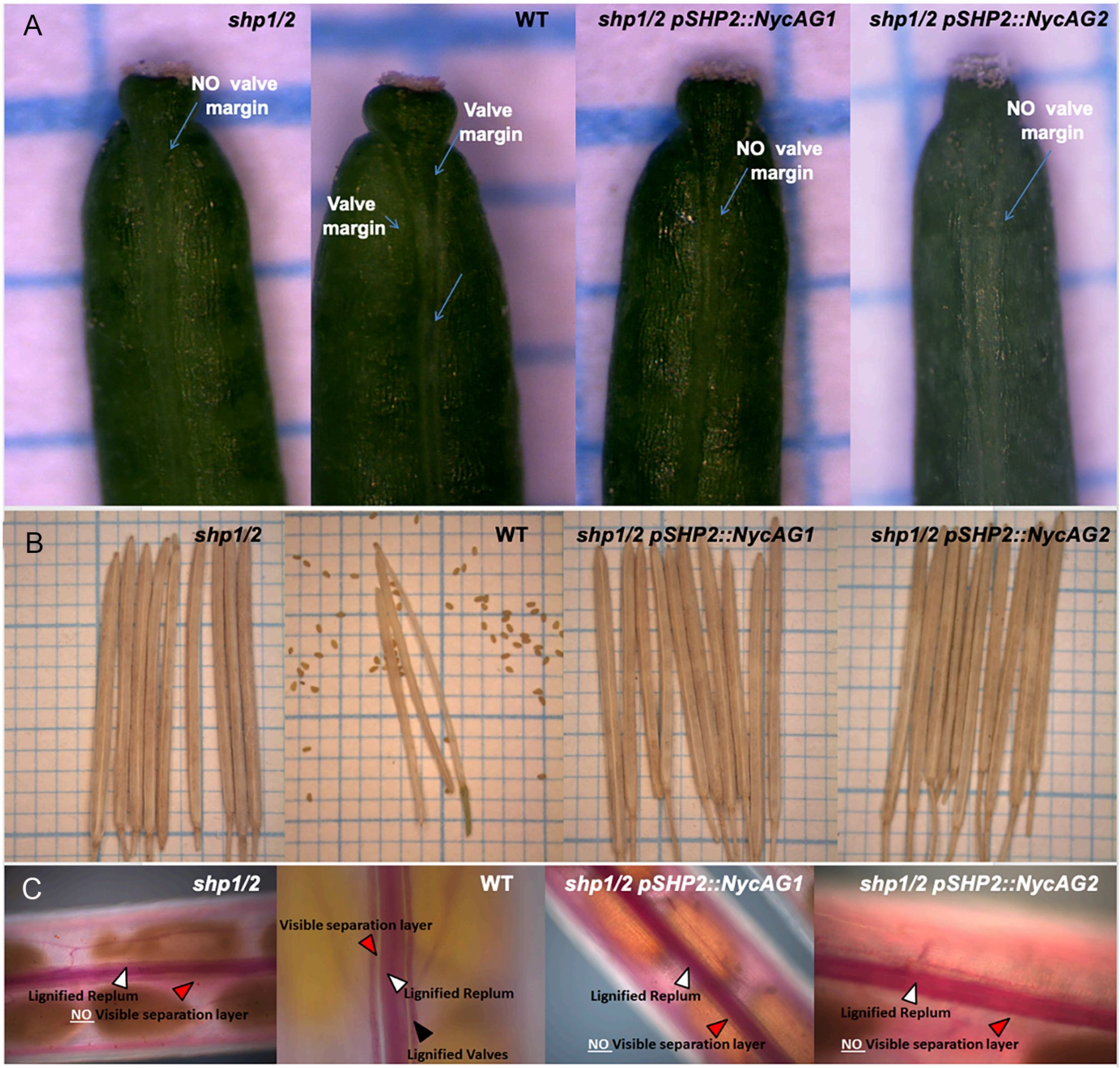

Supplementary Figure 9. *Arabidopsis* WT, *shp1 shp2*, *shp1/2::NycAG1* and *shp1/2::NycAG2* mutant fruit phenotypes. (**A**) Valve margin differentiation in *shp1 shp2*, WT, and *shp1/2::NycAG1* and *shp1/2::NycAG2*. (**B**) Separation phenotypes of the siliques. WT mature fruits show valve separation (fruit dehiscence) and seed abscission while neither the *shp1 shp2* double mutant nor *shp1/2::NycAG1* and *shp1/2::NycAG2* are able to dehisce and cannot abscise the seeds. (**C**) Lignification pattern in wild-type and *shp1 shp2* mutant plants. Fruits stained with phloroglucinol; magenta staining indicates lignin deposition (see methods). In WT silique, lignin is detected in the replum and valve margin and a visible separation layer can be observed. However, in *shp1 shp2* double mutant and in *shp1/2::NycAG1* and *shp1/2::NycAG2* fruits, no lignified valve margin was present and no separation layer was visible, consistent with no dehiscent phenotype upon maturity.
