# Supplementary material for: Expression and Functional Analyses of Nymphaea caerulea MADS-Box Genes Contribute to Clarify the Complex Flower Patterning of Water Lilies
Source: Front Plant Sci. 2021 Sep 22;12:730270. doi: 10.3389/fpls.2021.730270 (PMC8492926; doi:10.3389/fpls.2021.730270)
Supplement: Supplementary file 10 [file Data_Sheet_10.PDF]

```
                                       FUL motif                                                      farnesylation motif      euAP1 motif

euAP1
Heuchera_americana_euAP1               FLNPQP-------LPCLNIS-STYQ-------G------------GAQPEAMRRNELDLTLE-------PIYSCHLGCF-
Phytolacca_americana_euAP1             FLLPPP-------LTSWNTAGDAYH-------G-------------QIAGEVKRNDLDLTLE-------PVYSCXXGCF-
Corylopsis_sinensis_euAP1              FLLQQP-------LPCLNIG-NTYN-------QGV----------GGGAPEVRRNELDLTLE-------PVYPCHIGCFA
Solanum_lycopersicum_euAP1             FLLQPH-------Q-CLNMG-GNYQ-------D---------- ---EVAEARRNNELDLNLDSLY---PLYNMNKHL--
Syringa_vulgaris_euAP1                 FFLAPQ-------LPGLNIG-GAYE-------G-------------ESAEATRN-ELDLTLE-------PVYNC------

                                                                                                                     FUL-like motif
euFUL
Pisum_sativum_euFUL                    ALVPQP-------LETLNIG-CSPQ-------DRG-----------------DNEGS-QTQSN-----AL----LPHWMLRH---
Corylopsis_sinensis_euFUL              ILLS-QP------PQSLNIG-TY---------QTGC-------------SEGEEGTPFQQ—RPNT---L-----LPHWMVSHL-
Antirrhinum_majus_euFUL                AGAPQS-------LSSLSLS-EICQ-------GQRD-------------NNGEVEG---SRNQNQSSNKI----LPPWML-----
Petunia_x_hybrida_euFUL                FMFPPP-------PQSLLHSLTIG--------GNFQI------------GQENG-AQIR-----PNSNPL----MPPWMLRHVNQ
Heuchera_americana_euFUL               VLPSQP-------FQSVDTG-SSFQ-------TAG--------------NGGEVETS-TSLNR---TNSL----LPHWMVSHL--
Petunia_x_hybrida_euFUL                FVLSQP-------LNSLHLG-EAYP-------SAG----------------DNGEVEG--SSRQQ—PPNTV----MPPWMLRHLNG
Solanum_lycopersicum_euFUL             FVLPQQ-------LDSPHLG-EAYQ-------STNVI------------DNGEVEGGSSSQQQGAANNTV----MPPWML-----
Solanum_lycopersicum_euFUL2            FVLPHP-------FNNLHIG-ESIP-------KCR-------------RQWXSRRIIPATNN-KTVLLW-----MPPWML-----

FUL-like
Nymphaea_caerulea_NycFL                FLFNSHS-------PTPDSG--AYQ-------TRN---------------REAEESPHQ-HLQTQTSINN----MPPWTLRLMTE-
Nuphar_advena_AP1_partial              FLSTSHP-------HPALNV--RFE-------SRE---------------DRDEEELVEDHHHSLTQPSNVV---PPWLFH-----
Amborella trichopoda_CAL-like          FLLA-SP------LPTLNIG-TY---------HQGN---------------EVEEEGARPPA--RTNS—L----MPPWMVRHVNE-
Magnolia grandiflora AP1-like          FLLPSQS------LPSLNIG-GNYQ-------GRSN---------------GGGEEEASAAQP-RPIS-L----MPPWMLRHVNE-
Nigella_damascena_FUL-like1            FLFNGLK-------PSVDIG--SSH-------KVR---------------AEGGESPAQ-RLSAKDN-RS----MPPWMLRFVTQ-
Cabomba_caroliniana_AP1-like           FLL-----------PTLNIG-TY---------HRGN---------------EVEEEGARPPA--RTNS—L----MPSWMLC----
Magnolia_figo_FUL-like                 PLILLP-------PPALSXGSFRQE-------NGPS---------------VEG--EAAPR-VAQKNS-L----LPPWMX-----
Peperomia_caperata_FUL-like            TLFPIP-------LHTLSIG-YCKE-------DGKA-----------------IELGLENEPRPLSQK------MPPWML-----
Peperomia_caperata_FUL-like            VPLPDAV-------PALNTSEPTNQ-------SSGSX-------------G----EEEVXAQPSQTKT—L----MPPWMLHHL--
Ranunculus_bulbosus_FUL-like           VPLPDXV-------PALNTNGPTNQ-------SSGSG-------------GXGGGGEEVVAQPPQTKT—P----MPPWMLXHL--
Ranunculus_bulbosus_FUL-like           ---------------LNVSGTSTQ-------SSETQ-------------G-----EGDVVQPLRAKT—L----MPPWMLNHL--
Ranunculus_acris_FUL-like              FLL-SQS------LPNLNIGNGSYQ-------VRGG--------------NNGNEEEIRTQTT-RTNTATP---MPHWMVNHLR-
Papaver_nudicaule_FUL-like             FTL--QS------HPSLNIG-GNYQ-------GRSS----------------GREDE-VPQTQA-RPTI—L----MPSWMLC----
Papaver_nudicaule_FUL-like             FLLS-------QELPSLTICTGTYE-------PAARP-------------IAIQT---------------V---MPPWMLRHL--
Papaver_somniferum_FUL-like            FLL-SQS------LPSLNIGSGSYQ-------ARGG-------------DNGNEEGNRTQTT-RTNTATL----MPPWMLN----
Papaver_somniferum_FUL-like            FLT--------QALPSLTLRTGYYQ-------TARVV-------------VCEEDGARSNM---------X---MPPWML-----
Chelidonium_majus_FUL-like             FLL-SQS------LPSLNIG-GSYH-------MRGS-------------NGSEEEGVRPQTT-RTNT-TL----MPPWMVHHL---
```

Supplementary Figure 10. Partial alignment of some representative euAP1, euFUL and AP1/FUL-like proteins, respectively, to evidence the different FUL, euAP1 and paleoAP1 motifs. NycFL is indicated in bold. The regions of interest are boxed to show the relative aminoacid residues. Sequences have been selected according to Shan *et al.* (2007) and Pabon-Mora *et al.* (2012).
