# Supplementary material for: Expression and Functional Analyses of Nymphaea caerulea MADS-Box Genes Contribute to Clarify the Complex Flower Patterning of Water Lilies
Source: Front Plant Sci. 2021 Sep 22;12:730270. doi: 10.3389/fpls.2021.730270 (PMC8492926; doi:10.3389/fpls.2021.730270)
Supplement: Supplementary file 11 [file Data_Sheet_11.PDF]

Supplementary File 1. Statistical analysis of the expression levels of the *NycACT* reference gene in all the flower and fruit tissues analyzed in this work.
Non paramentric Kruscal-Wallis tests were performed in order to asses the uniform expression of the used reference gene and the results are reported below.
The tests were performed directly on the Ct values obtained by qPCR experiments, using the default parameter of the XLSTAT software. Bonferroni corrected significance levels are calculated by the algorithm.

<u>Comparison of *NycACT* expression levels in Early Stage tissues</u>

Kruskal-Wallis test:

K (Observed value)	34.847
K (Critical value)	16.919
DF	9
p-value (Two-tailed)	< 0,0001
alpha	0.05

An approximation has been used to compute the p-value.

Test interpretation:
H0: The samples come from the same population.
Ha: The samples do not come from the same population.

As the computed p-value is lower than the significance level alpha=0,05, one should reject the null hypothesis H0, and accept the alternative hypothesis Ha.

The risk to reject the null hypothesis H0 while it is true is lower than 0,01%.

Ties have been detected in the data and the appropriate corrections have been applied.

Multiple pairwise comparisons using Dunn's procedure / Two-tailed test:
p-values:

	SEPALS	OUTER PETALS	INNER PETALS	PETALOID STAMENS TIPS	PETALOID STAMENS ANTHERS	INNER STAMENS	CARPELS	OVULES	FRUIT	ARILS
SEPALS	1	0.7390	0.0581	0.8540	0.8994	0.6544	0.0375	0.6886	0.6516	0.1198
OUTER PETALS	0.7390	1	0.0584	0.5854	0.8160	0.3562	0.0344	0.4704	0.8506	0.1435
INNER PETALS	0.0581	0.0584	1	0.0358	0.0438	0.0076	0.8325	0.0426	0.1633	0.8656
PETALOID STAMENS TIPS	0.8540	0.5854	0.0358	1	0.7410	0.8076	0.0223	0.8092	0.5251	0.0820
PETALOID STAMENS ANTHERS	0.8994	0.8160	0.0438	0.7410	1	0.5128	0.0259	0.5901	0.7080	0.1098
INNER STAMENS	0.6544	0.3562	0.0076	0.8076	0.5128	1	0.0039	0.9559	0.3428	0.0298
CARPELS	0.0375	0.0344	0.8325	0.0223	0.0259	0.0039	1	0.0289	0.1142	0.7227
OVULES	0.6886	0.4704	0.0426	0.8092	0.5901	0.9559	0.0289	1	0.4285	0.0805
FRUIT	0.6516	0.8506	0.1633	0.5251	0.7080	0.3428	0.1142	0.4285	1	0.2697
ARILS	0.1198	0.1435	0.8656	0.0820	0.1098	0.0298	0.7227	0.0805	0.2697	1

Bonferroni corrected significance level: 0,0011

Significant differences:

	SEPALS	OUTER PETALS	INNER PETALS	PETALOID STAMENS TIPS	PETALOID STAMENS ANTHERS	INNER STAMENS	CARPELS	OVULES	FRUIT	ARILS
SEPALS	No	No	No	No	No	No	No	No	No	No
OUTER PETALS	No	No	No	No	No	No	No	No	No	No
INNER PETALS	No	No	No	No	No	No	No	No	No	No
PETALOID STAMENS TIPS	No	No	No	No	No	No	No	No	No	No
PETALOID STAMENS ANTHERS	No	No	No	No	No	No	No	No	No	No
INNER STAMENS	No	No	No	No	No	No	No	No	No	No
CARPELS	No	No	No	No	No	No	No	No	No	No
OVULES	No	No	No	No	No	No	No	No	No	No
FRUIT	No	No	No	No	No	No	No	No	No	No
ARILS	No	No	No	No	No	No	No	No	No	No

Comparison of *NycACT* expression levels in Late Stage tissues

Kruskal-Wallis test:

K (Observed value)	34.847
K (Critical value)	16.919
DF	9
p-value (Two-tailed)	< 0,0001
alpha	0.05

An approximation has been used to compute the p-value.

Test interpretation:
H0: The samples come from the same population.
Ha: The samples do not come from the same population.

As the computed p-value is lower than the significance level alpha=0,05, one should reject the null hypothesis H0, and accept the alternative hypothesis Ha.

The risk to reject the null hypothesis H0 while it is true is lower than 0,01%.

Ties have been detected in the data and the appropriate corrections have been applied.

Multiple pairwise comparisons using Dunn's procedure / Two-tailed test:

p-values:

	SEPALS	OUTER PETALS	INNER PETALS	PETALOID STAMENS TIPS	PETALOID STAMENS ANTHERS	INNER STAMENS	CARPELS	OVULES	FRUIT	ARILS
SEPALS	1	0.2634	0.1317	0.3950	0.2709	0.2322	0.0036	0.0086	0.0038	0.0061
OUTER PETALS	0.2634	1	0.5501	0.7253	0.9471	0.9587	0.0035	0.0253	0.0080	0.0150
INNER PETALS	0.1317	0.5501	1	0.3595	0.4813	0.5363	0.0141	0.0615	0.0235	0.0414
PETALOID STAMENS TIPS	0.3950	0.7253	0.3595	1	0.7592	0.6660	0.0023	0.0160	0.0050	0.0093
PETALOID STAMENS ANTHERS	0.2709	0.9471	0.4813	0.7592	1	0.8964	0.0015	0.0189	0.0052	0.0101
INNER STAMENS	0.2322	0.9587	0.5363	0.6660	0.8964	1	0.0011	0.0199	0.0051	0.0103
CARPELS	0.0036	0.0035	0.0141	0.0023	0.0015	0.0011	1	0.8077	0.6467	0.8180
OVULES	0.0086	0.0253	0.0615	0.0160	0.0189	0.0199	0.8077	1	0.8865	0.9747
FRUIT	0.0038	0.0080	0.0235	0.0050	0.0052	0.0051	0.6467	0.8865	1	0.8505
ARILS	0.0061	0.0150	0.0414	0.0093	0.0101	0.0103	0.8180	0.9747	0.8505	1

Bonferroni corrected significance level: 0,0011

Significant differences:

	SEPALS	OUTER PETALS	INNER PETALS	PETALOID STAMENS TIPS	PETALOID STAMENS ANTHERS	INNER STAMENS	CARPELS	OVULES	FRUIT	ARILS
SEPALS	No	No	No	No	No	No	No	No	No	No
OUTER PETALS	No	No	No	No	No	No	No	No	No	No
INNER PETALS	No	No	No	No	No	No	No	No	No	No
PETALOID STAMENS TIPS	No	No	No	No	No	No	No	No	No	No
PETALOID STAMENS ANTHERS	No	No	No	No	No	No	No	No	No	No
INNER STAMENS	No	No	No	No	No	No	No	No	No	No
CARPELS	No	No	No	No	No	No	No	No	No	No
OVULES	No	No	No	No	No	No	No	No	No	No
FRUIT	No	No	No	No	No	No	No	No	No	No
ARILS	No	No	No	No	No	No	No	No	No	No

Comparison of *NycACT* expression levels among Early stage and Late stage tissues

Kruskal-Wallis test:

K (Observed value)	159.860
K (Critical value)	30.1435
DF	19
p-value (Two-tailed)	< 0,0001
alpha	0.05

An approximation has been used to compute the p-value.

Test interpretation:
H0: The samples come from the same population.
Ha: The samples do not come from the same population.

As the computed p-value is lower than the significance level alpha=0,05, one should reject the null hypothesis H0, and accept the alternative hypothesis Ha.

The risk to reject the null hypothesis H0 while it is true is lower than 0,01%.

Ties have been detected in the data and the appropriate corrections have been applied.

Multiple pairwise comparisons using Dunn's procedure / Two-tailed test:

Early stage		Late stage	p-values:	Significant differences:
SEPALS	vs	SEPALS	0.001424338	No
OUTER PETALS	vs	OUTER PETALS	0.005156403	No
INNER PETALS	vs	INNER PETALS	0.044486229	No
PETALOID STAMENS TIPS	vs	PETALOID STAMENS TIPS	0.001990415	No
PETALOID STAMENS ANTHERS	vs	PETALOID STAMENS ANTHERS	0.003236101	No
INNER STAMENS	vs	INNER STAMENS	0.014588061	No
CARPELS	vs	CARPELS	0.027734113	No
OVULES	vs	OVULES	0.013901585	No
FRUIT	vs	FRUIT	0.811552821	No
ARILS	vs	ARILS	0.618076505	No

Bonferroni corrected significance level: 0,0003
