# Supplementary material for: Expression and Functional Analyses of Nymphaea caerulea MADS-Box Genes Contribute to Clarify the Complex Flower Patterning of Water Lilies
Source: Front Plant Sci. 2021 Sep 22;12:730270. doi: 10.3389/fpls.2021.730270 (PMC8492926; doi:10.3389/fpls.2021.730270)
Supplement: Supplementary file 12 [file Table_1.pdf]

Supplementary Table 1. Transcripts isolated and studied in this work. For each transcript, length (bp) and NCBI accession number are provided.

Transcript Name	NCBI Accession Number	Length (bp)
NycFL	MN626366	924
NycAP3-1	MN626367	800
NycAP3-2	MN626368	929
NycAP3-3	MN626386	1245
NycPI	MN626369	972
NycAG1	MN626373	1238
NycAG2	MN626374	1162
NycSTK	MN626375	787
NycAGL6	MN626370	1447
NycSEP	MN626371	1223
NycACT	MN626379	901
