# Supplementary material for: Expression and Functional Analyses of Nymphaea caerulea MADS-Box Genes Contribute to Clarify the Complex Flower Patterning of Water Lilies
Source: Front Plant Sci. 2021 Sep 22;12:730270. doi: 10.3389/fpls.2021.730270 (PMC8492926; doi:10.3389/fpls.2021.730270)
Supplement: Supplementary file 13 [file Table_2.pdf]

Supplemental Table 2. List of oligonucleotides used in this work.

All the oligonucleotides have been designed in this work except where differently indicated.

Primer name	5'-3' sequence	Gene name	Reference
**RT-PCR**			
**Oligo-dT primers**			
Right_PolyT_ORTOLAB	CCGGATCTCTAGACGGCCGCTTTTTTTTTTTTTTTTVN		
**RT-PCR Fw primers**			
MADS_deg_K98	GGGGTACCAAYMGICARGTIACITAYTCIAAGMGIMG		Kramer et al. 1998
MADS_deg_K04	GGIMGIGGIAARATIGARATIAARMGIAT		Kramer et al. 2004
MADS_deg2_S04	ATGGSIMGIGGIAARATISARAT		Stellari et al. 2004
1left_ORTOLAB	AAGAAGGCVWASGAGYTSWCYG		
2left_ORTOLAB	AAGAAGGCVWASGAGYTSWCYGT		
3left_ORTOLAB	AAGAAGGCVWASGAGYTSWCYGTS		
1aleft_ORTOLAB	AYAAACTMMRGVAGMAGAWC		
2aleft_ORTOLAB	AYAAACTMMRGVAGMAGAWCR		

**RT-PCR Rev primer**		
Right_PCR_ORTOLAB	CCGGATCTCTAGACGGCCGCTT	
**qPCR primers**		
Nc_AP1_c1_for	CAGAGGGGGACACTGTGGTA	*NycFL*
Nc_AP1_c1_rev	GCTGTGTCTTCCTCAGTCGAA	
Nc_AP3-I_c2_for	AGGTTAGCTGATGACCCTAGAA	*NycAP3-1*
Nc_AP3-I_c2_rev	CACACACTGTGCCAGTGAAA	
Nc_AP3-II_c1_for	TGGGCTGTCGAAATCCAACAT	*NycAP3-2*
Nc_AP3-II_c1_rev	CTTCTTGTAACTCCCTGAAACCT	
Nc_AP3-III_for	GATTCTTGATGCAGATAAGGAG	*NycAP3-2*
Nc_AP3-III_c2_rev	CACACACTGTGCCAGTGAAA	
Nc_PI_c1_for	AGAGAATGCCTCCAACAGGC	*NycPI*
Nc_PI_c1_rev	CATTCGGTCTGCTTCATGCG	
Nc_AG1_c6_for	AGAACGAAAGAGCGGAGCAG	*NycAG1*
Nc_AG1_c6_rev	TTGGTGCGAGTATTGGTGGT	
Nc_AG2_c1_for	AGAACACGACTAACCGGCAG	*NycAG2*
Nc_AG2_c1_rev	CCCAAGTCGCTCATCCAAGT	

Nc_AG3_5'	CAAGAAGGCGTGCGCTGAAA	*NycSTK*
Nc_AG3_3'	CTGGTTATGCCCCGCTCTATTTT	
Nc_AGL6_c10_for	AGACTTTGCTGATGGGGCAA	*NycAGL6*
Nc_AGL6_c10_rev	GGCAGACACGGCCAGTAAT	
Nc_SEP1_c2_for	TTCTGTACTCTGCGATGCCG	*NycSEP*
Nc_SEP1_c2_rev	AGGACCCAGATCTTCACCGA	
**Cloning of *N. caerulea* genes for *A. thaliana* complementation**		
ninph001_GW	GGGGACAAGTTTGTACAAAAAAGCAGGCTTCATGGGGAGGGGAAAGATCG	*NycAG1*
ninph002_GW	GGGGACCACTTTGTACAAGAAAGCTGGGTCGTCATCCAAGTTGCAAGGCAG	
ninph003_GW	GGGGACAAGTTTGTACAAAAAAGCAGGCTTCGGGAATTTGGAGAAGATGGGAAG	*NycAG2*
ninph004_GW	GGGGACCACTTTGTACAAGAAAGCTGGGTCTCTTGGATCCTTCCCAAGTCG	
**Genotyping of *A. thaliana* transgenic lines**		
ninph007	GCTGAAACGAATGCGCAGTA	*NycAG1*
ninph008	TACTGCGCATTCGTTTCAGC	
ninph009	AGTGAAATGAGTCACCGAGACC	*NycAG2*
ninph010	GGTCTCGGTGACTCATTTCACT	

AtP_1805_FW	CTTCACATCATGTGTCACATTAAGTTGC	*promAG*
AtP_4304_FW	AGAATAGTATCTTTAGGCCAATGAACAAAT	*promSHP*
AtP_1665_REV_int	GAACTACTCACACATTATTCTGG	*T35S*
**Transgene expression quantification**		
nymAG1_for	AGAACGAAAGAGCGGAGCAG	*NycAG1*
nymAG1_rev	TTGGTGCGAGTATTGGTGGT	
nymAG2_for	AGAACACGACTAACCGGCAG	*NycAG2*
nymAG2_rev	CCCAAGTCGCTCATCCAAGT	
Actin RT 861_ for	CTCAGGTATTGCAGACCGTATGAG	*Arabidopsis actin8*
Actin RT 862_rev	CTGGACCTGCTTCATCATACTCTG	
**Cloning of *N. caerulea* genes for Y2H assay**		
attB1_for_SEP	GGGGACAAGTTTGTACAAAAAAGCAGGCTCAGGATGGGAAGAGGCAGAGT	*NycSEP*
attB2_rev_SEP	GGGGACCACTTTGTACAAGAAAGCTGGGTTCATACTAGCCACGGAGGGA	
attB1_for_AGL6	GGGGACAAGTTTGTACAAAAAAGCAGGCTCGGGTTGTGCCTTGAGAGATCA	*NycAGL6*
attB2_rev_AGL6	GGGGACCACTTTGTACAAGAAAGCTGGGTAAGGATTACAGGACCCAGCCT	
attB1_for_PI	GGGGACAAGTTTGTACAAAAAAGCAGGCTCAGTGGTCGGTTGCTGGAGAT	*NycPI*

attB2_rev_PI	GGGGACCACTTTGTACAAGAAAGCTGGGTAGTGCATCAAACCAATGCCC	
attB1_for_AP3	GGGGACAAGTTTGTACAAAAAAGCAGGCTCATGGGTCGTGGTAAGATA	*NycAP3-1*
attB2_rev_AP3_1	GGGGACCACTTTGTACAAGAAAGCTGGGTTCATCCTAGACTTAAATCATG	
attB1_for_AP3	GGGGACAAGTTTGTACAAAAAAGCAGGCTCATGGGTCGTGGTAAGATA	*NycAP3-2/3*
attB2_rev_AP3_2	GGGGACAAGTTTGTACAAGAAAGCTGGGTTTAATCCATGCCCACAAAATA	
attB1_for_AG3	GGGGACAAGTTTGTACAAAAAAGCAGGCTCATGGGAAGGGGAAAGATTGAGATC	*NycSTK*
attB2_rev_AG3	GGGGACCACTTTGTACAAGAAAGCTGGGTGGAGCATACAATTCAGACAACAC	
attB1_for_AG2	GGGGACAAGTTTGTACAAAAAAGCAGGCTCGGGAATTTGGAGAAGATGGGAAGAG	*NycAG2*
attB2_rev_AG2	GGGGACCACTTTGTACAAGAAAGCTGGGTCTACATAAGATTGGGACCTTGATC	
attB1_for_AG1	GGGGACAAGTTTGTACAAAAAAGCAGGCTCAGCAAACTGAGGAAGATGGGGA	*NycAG1*
attB2_rev_AG1	GGGGACCACTTTGTACAAGAAAGCTGGGTTGAGGCTTTGGGCTTTCAATG	
attB1_for_AP1/FUL	GGGGACAAGTTTGTACAAAAAAGCAGGCTCCTCTGCGACGCCGAGGTTGC	*NycFL*
attB2_rev_AP1/FUL	GGGGACCACTTTGTACAAGAAAGCTGGGTTCATATGAGCTGAAGGGTCCAGGG	
